# Cognitive profiles of paedophilic behaviour: a meta-analytic and systematic review of developmental vs acquired forms

**DOI:** 10.3389/fpsyt.2025.1568244

**Published:** 2025-06-09

**Authors:** Cristiano Costa, Lucia Ronconi, Stefano Ferracuti, Alexa Schincariol, Cristina Scarpazza

**Affiliations:** ^1^ Department of General Psychology, School of Psychology, University of Padua, Padua, Italy; ^2^ Department of Human Neurosciences, Faculty of Medicine and Dentistry, Sapienza University of Rome, Rome, Italy; ^3^ Neuroscience Center, Department of General Psychology, School of Psychology, University of Padua, Padua, Italy; ^4^ Istituto di Ricovero e Cura a Carattere Scientifico (IRCCS) S. Camillo Hospital, Venice, Italy

**Keywords:** child sexual offending, pedophilia, pedophilic disorder, cognitive profile, impulsivity, social cognition, meta-analysis, systematic review

## Abstract

**Background:**

Developmental and acquired paedophilic behaviour are considered two distinct phenomena, yet no study has systematically compared the cognitive profiles of individuals committing these forms of child sexual offenses (CSO). This study explored whether individuals with developmental and acquired paedophilic behaviour are characterised by similar or different neuropsychological underpinnings and how these differences manifest themselves in observable behaviour.

**Methods:**

Thirty-four studies on developmental CSO and 17 (describing 21 cases) on acquired CSO were included. Multivariate meta-analytic approaches were adopted to investigate the cognitive abilities of individuals who committed CSO with (P+CSO) and without (CSO) a diagnosis of paedophilia (P), while a systematic review was conducted to identify the cognitive features of acquired CSO.

**Results:**

Meta-analytic findings showed overall worse neuropsychological performances for developmental CSO compared to the control group (*μ* = −0.186; *p* = .002). Subgroup analyses confirmed these results for both CSO (*μ* = −0.232; *p* <.05) and P+CSO (*μ* = −0.153; *p* <.05). The systematic review on acquired CSO revealed that all individuals (100%) exhibited deficits in inhibitory control and 62.5% of them showed concomitant impairments in social-cognition abilities.

**Conclusions:**

Developmental and acquired paedophilic behaviours share inhibitory control deficits, even though with different characteristics; however, social-cognitive deficits appear specific to acquired CSO. These findings provide insights into the neurocognitive underpinnings of these behaviours, highlighting distinct mechanisms that may influence their *modi operandi*.

## 1 Introduction

Sexual offenses against children are a major public concern affecting numerous individuals (1) and causing significant trauma and human suffering for victims and their families (2). Although child sexual offending is often considered analogous with paedophilia, this represents a fundamental misuse of terminology.

The fifth version of the Diagnostic and Statistical Manual of Mental Disorders (5th ed.; DSM–5; 3) made for the first time a clear distinction between paedophilia and paedophilic disorder: while paedophilia is defined as persistent sexual attraction to children, paedophilic disorder can be diagnosed only when paedophilia is accompanied by recurrent, intense sexual arousal or urges involving sexual activities with children, when they cause marked distress in the individual, or result in sexual offenses against children (4, 5). Importantly, not all individuals with paedophilia commit sexual offenses against children. Indeed, some of them manage to confine their sexual urges and desires to fantasies about sexual contact with children (6–10), whereas others proactively seek therapeutic interventions to manage overwhelming impulses before acting on them (6, 10–12).

Traditionally, research has focused on individuals whose paedophilic interests emerge in adolescence and persist over time. However, a growing body of literature describes cases in which sexual interest in children arises *de novo* in adulthood, typically in the context of an identifiable neurological insult - such as a tumour, traumatic brain injury, or neurodegenerative disease (13–15)^1^. These cases, to which we refer as *acquired* CSO, raise new questions about the neurobiological roots of such behaviour.

In this paper, we focus on individuals who have committed child sexual offenses (CSO), regardless of whether they meet formal diagnostic criteria for paedophilia. We distinguish between two clinical manifestations: (1) *developmental* CSO, encompassing both individuals with paedophilic disorder (P+CSO)^2^ and those without (CSO), whose offending behaviour is not associated with a known neurological event; and (2) *acquired* CSO, referring to individuals whose paedophilic urges and offending behaviour arise in direct relation to neurological damage.

Given the clinical and theoretical significance of this distinction, the present study pursues two main objectives. First, we examine differences within the developmental CSO group – comparing P+CSO and CSO individuals – to determine whether observed cognitive impairments are more strongly associated with paedophilic preference or with the act of offending itself. Second, we qualitatively compare developmental and acquired CSO to assess whether they reflect distinct neuropsychological profiles, as suggested by their divergent aetiologies, neural substrates, and behavioural features (21–23). By addressing both levels of analysis, this study offers a comprehensive framework to understand the cognitive mechanisms underlying child sexual offending, with implications for diagnosis, treatment, and legal accountability.

Regarding acquired CSO, some researchers argue that individuals with this condition are usually characterised by an underlying sexual interest in children that can be unmasked by a change in the baseline functioning – like a neurological insult – impairing their ability to regulate pre-existing tendencies (24, 25). This interpretation aligns with cases where premorbid sexual interest in children was documented (25). However, other cases challenge this hypothesis (26). For instance, some individuals with acquired CSO deny any prior attraction to children. In these cases, it has been suggested that functional impairments, such as damage to the hypothalamus, may lead to a shift in sexual orientation (26). Indeed, a recent review showed that only 19% of the published cases of individuals with acquired CSO had premorbid interests in children (27), highlighting that the hypothesis based on the unmasking of previous paedophilic tendencies may not fully explain the phenomenon. Said review suggests that acquired CSO might be one of many symptoms of a general disinhibition syndrome following basal frontotemporal damage, or at least of a hypersexuality-related disorder induced by subcortical damage. Whether dis-inhibition is sufficient to cause the onset of acquired CSO is still not known. Research has been scant, but individuals with acquired CSO typically manifest a behavioural fracture resulting in sexual offenses against children (10, 13, 28–30).

Developmental and acquired CSO seem to therefore represent two distinct conditions (21). The key distinction lies in their nature: developmental CSO is often associated with paedophilia, resulting in a paedophilic disorder (3). Multiple theoretical models have been proposed to explain paedophilic disorder, which can generally be categorized into three main approaches: (1) theories highlighting paedophilic disorder’s predetermined and/or unchangeable nature (i.e., evolutionary; 31) or genetic accounts (32); (2) those emphasizing its neurodevelopmental nature (33); (3) and those emphasizing a multifactorial explanation (34–36). In contrast, acquired CSO occurs *de novo*, as a symptom of an underlying medical condition (21, 29, 37, 38). In fact, individuals with acquired CSO are usually characterized by a late onset of paedophilic urges, which arises independently of their developmental trajectory (13, 28). This distinction has important implications for treatment. In cases of developmental CSO, pharmacological or non-pharmacological treatments should focus on managing paedophilia itself – when present – (39, 40). Conversely, treatment for acquired CSO should target the underlying condition (13, 26).

From a neuropsychological perspective, individuals with developmental paedophilic disorder present a complex cognitive profile that has been relatively well characterised, though findings remain mixed and are constrained by some methodological limitations, most notably the inclusion of heterogeneous offender samples, that often conflate individuals with and without a formal diagnosis of paedophilia. Overall, intellectual functioning tends to fall within the average range, with most studies reporting no significant IQ differences between paedophilic and non-paedophilic sexual offenders (41–44). Executive functions have been a key focus of investigation. While working memory, set-shifting, and planning abilities are generally preserved, some studies suggest relative weaknesses in processing speed, verbal fluency, and inhibitory control (see 43 for a summary). Recent findings by Picard et al. (43) further refine this profile by comparing the cognitive performance of 58 men convicted of various sexual offenses (including contact sexual offenses, non-contact sexual offenses, and child sexual abuse material), 20 of whom were diagnosed with paedophilic disorder. Interestingly, those with paedophilic disorder outperformed non-paedophilic offenders on tasks assessing verbal memory and visual discrimination, but made more errors on a set-shifting task, suggesting subtle difficulties with cognitive flexibility. Notably, all groups performed within the normative range across cognitive domains, pointing to mild rather than overt cognitive impairments (43).

By contrast, the neuropsychological profile of individuals with acquired CSO remains poorly defined by systematic research and is primarily reconstructed from individual case studies. Nevertheless, a consistent pattern emerges across cases, typically involving impairments in executive functions – particularly in impulse control, social cognition, and moral reasoning – which typically reflect the nature and localisation of the underlying organic pathology. For example, Burns and Swerdlow (13) described a patient with an orbitofrontal tumour who developed paedophilic behaviour alongside disinhibition, impaired moral judgement, and poor insight, all of which resolved after tumour resection. Similarly, Sartori et al. (26) reported a case involving compression of the hypothalamus and the orbitofrontal cortex by a *Clivus Chordoma*, where impulsive sexual offences occurred within the broader context of dysexecutive syndrome and diminished social and moral awareness. These deficits were not only evident in formal neuropsychological testing but also manifested in daily life, as observed by relatives and clinicians. Taken together, these findings underscore the aetiological and clinical divergence between developmental and acquired CSO, suggesting that similar behaviours may be underpinned by fundamentally distinct neuropsychological mechanisms.

The neural bases of developmental and acquired CSO are also distinct and reflect the different nature of these two forms of offending behaviour. Structural alterations in developmental P+CSO are observed in both grey (45) and white matter (7). While findings are heterogeneous, one result replicated across multiple studies is reduced right amygdala volume in individuals with P+CSO compared to controls (46–48). Regarding functional alterations, studies have suggested that brain activity is not generally altered in developmental CSO but deviates specifically in response to sexual stimuli (49–55). These alterations are primarily localised in the left anterior insular cortex, the left claustrum, and the anterior midcingulate cortex – key hubs of brain networks regulating sexual arousal (56). In contrast, acquired CSO, being a consequence of an underlying neurological condition, is characterized by evident brain lesions or alterations that may be the result of various causes, including traumatic (38, 57), neoplastic (13), surgical (28), degenerative (58), or demyelinating origin (14, 27, 37).

Overall, the neural basis of developmental and acquired CSO described in the literature seem spatially heterogeneous, hampering a clear understanding of the neural origin of these offending behaviours (15). However, a critical distinction emerges when comparing the two conditions. A recent meta-analysis by Scarpazza et al. (59) investigating structural and functional neuroimaging studies in individuals with developmental paedophilia failed to identify consistent brain alterations, even when applying liberal statistical thresholds. The findings revealed high variability in the localization of reported brain changes, with some clusters emerging only under exploratory analyses, such as in the middle cingulate, superior frontal, and occipital gyri. This lack of convergence suggests that developmental CSO is likely underpinned by distributed and subtle neural alterations that do not consistently affect the same anatomical and functional substrates across individuals. Such heterogeneity may reflect the multifactorial aetiology of paedophilic disorder, encompassing genetic, neurodevelopmental, and psychosocial components (36), and highlights the challenges of identifying a single neurobiological model to account for this condition. In contrast, for acquired CSO, progress has been made in linking behaviour to specific neural networks. Although brain lesions in individuals with acquired CSO are also spatially heterogeneous, by means of lesion mapping analysis Scarpazza, Finos et al. (23) identified a shared brain network consistently damaged in all cases included in the analysis. This network includes the orbitofrontal cortex bilaterally and posterior midline structures, such as the posterior cingulate cortex and praecuneus. Moreover, Joyal (27) highlighted the potential influence of damage to the basal frontotemporal regions as well, including basal temporal areas, further enriching our understanding of the neural basis of acquired CSO. This convergence onto specific neural networks for acquired CSO has been supported by a functional characterization approach (60, 61). This method links brain regions to the psychological functions they are most likely to underlie based on patterns of experimental activation derived from the literature. While this approach was not applicable to developmental CSO due to inconsistent neural findings, its application to acquired CSO has provided significant insights, linking the orbitofrontal cortex to action inhibition and the posterior midline structures to social cognition, specifically to the construct of theory of mind, the ability to understand the social and moral disvalue of one’s actions, and the capacity to discriminate right from wrong (23). These findings underscore that acquired CSO may be traced back to deficits in specific neural networks, particularly those supporting inhibitory control and social cognition (23). Such impairments likely diminish the ability to regulate inappropriate urges and undermine moral and social awareness, thereby contributing to offending behaviours. This stands in contrast to developmental CSO, where the lack of spatially convergent neural alterations suggests a more heterogeneous and sparse neurobiological basis, complicating efforts to establish a coherent brain-behaviour model.

The presence of cognitive difficulties in individuals with acquired CSO is reflected in their *modus operandi*, which is described as impulsive and disorganized in the literature. Offenses are often characterized by lack of premeditation, an absence of attempts to mask the behaviour, and a tendency toward spontaneous confession (62). These traits align with the findings from three major reviews on acquired CSO (14, 27, 59) which conclude that in the majority of the reviewed cases (82% according to 27), acquired paedophilic behaviours involve generalized behavioural impulsivity, including hypersexuality. In contrast, the *modus operandi* of individuals with developmental CSO suggests intact inhibition and social cognition, as individuals show some degree of voluntary behavioural control (63–65), and offenses are typically premeditated, and there is often an attempt to induce the victim to silence about the crime (63, 66, 67). This behavioural pattern has led to developmental CSO being conceptualized as a compulsive rather than an impulsive disorder (63).

Although the neuropsychological underpinnings of individuals with developmental CSO have been widely studied in recent years (68, 69), inconsistencies in the literature remain regarding the cognitive profiles of these individuals, particularly in the domain of impulsivity, as demonstrated by contradictory findings across studies (70, 71). Notwithstanding the advances in knowledge achieved so far, it remains unclear whether the two forms of CSO differ regarding the cognitive profile (15, 21, 36, 62).

To address these gaps, in the current study we investigated the cognitive profile of individuals who committed sexual offenses against children, focusing on whether neuropsychological differences can explain their behaviours. Specifically, we aimed to determine whether individuals who exhibited developmental CSO and individuals with acquired CSO are characterized by similar or different cognitive/neuropsychological underpinnings.

On the one hand, for developmental CSO, we conducted a multivariate and mixed-effect meta-regression meta-analyses on 34 studies, incorporating data from individuals convicted for CSO with a formal diagnosis of paedophilia (P+CSO) and those without (CSO). Moreover, by analysing these groups separately, we sought to clarify whether any observed cognitive deficits were more strongly linked to paedophilia itself or to committing child sexual abuse in general. On the other hand, for acquired CSO we performed a systematic review due to the reliance on case reports rather than group-level studies. This involved extracting detailed information on the cognitive abilities of individuals and quantifying the prevalence of specific impairments across cases.

By integrating quantitative and qualitative approaches, this study provides a comprehensive framework to investigate the neuropsychological mechanisms underlying developmental and acquired CSO. This analysis not only addresses inconsistencies in the literature but also offers insights into how distinct cognitive profiles might influence the behaviours associated with this form of offending.

## 2 Materials and methods

### 2.1 Meta-analysis on developmental CSO

#### 2.1.1 Search strategy

Following PRISMA Guidelines (72), an in-depth search was conducted on PubMed (up to October 2022) using the following terms:

(pedophilia) OR (idiopathic pedophilia) OR (developmental pedophilia) OR (pedophilic behavio*) OR (child sex* offen*) OR (child molest*) OR (sex offen* against child*)) AND ((neuropsychologic test) OR (executive functions) OR (cognition) OR (impaired cognition)).

Medical Subject Headings (MeSH) searches were also performed. Abstracts and titles were screened, followed by full-text evaluation against inclusion and exclusion criteria. Lastly, reference lists of relevant reviews and meta-analyses were inspected to identify additional studies.

#### 2.1.2 Inclusion and exclusion criteria

Studies were included if they met the following criteria: (a) studies presenting original data on the cognitive/neuropsychological performance of individuals who committed sexual offenses toward children, either with (P+CSO) or without (CSO) a formal diagnosis of paedophilia; (b) studies including a matched control group of healthy subjects or individuals who did not commit sexual offenses against children, or individuals with paedophilia or paedophilic disorder, but without a history of child sexual offenses (P-CSO); and (c) studies published in peer-reviewed journals in English.

Studies were excluded if they: (a) were reviews, meta-analyses, instrument validation studies, or case reports; (b) analysed mixed and/or heterogenous groups together, such as P+CSO, CSO without paedophilia, and P-CSO; (c) lacked a comparison group; or (d) did not provide minimal statistical data to compute effect sizes (e.g., sample size, mean, standard deviation).

#### 2.1.3 Data selection and extraction

Data extracted from each paper included sample size, mean (M), and standard deviation (SD) for cognitive and neuropsychological performance across the following domains: set switching, planning/reasoning, memory, attention, working memory, verbal fluency, verbal semantic, abstraction, social cognition, and inhibition. The choice to analyse these specific cognitive functions reflects the neuropsychological batteries and tests commonly used to assess the populations of interest in clinical and scientific settings.

Data extraction was conducted by the first author (CC) and independently checked by the last author (CS).

#### 2.1.4 Statistical analysis

To calculate effect sizes and standard errors for each observation, we utilised the *metafor* package (v. 3.4.0) in R (v. 4.1.2) (73). A random-effects meta-analysis was conducted to estimate weighted average effect sizes (g). The present meta-analysis involved observed outcomes/effect size estimates that cannot be assumed to be independent, because some studies contributed with multiple effect sizes due to the administration of multiple neuropsychological tests; therefore, the sampling errors in such studies are dependent and represent correlated effects (74). To address this, a variance–covariance matrix of the dependent estimates was constructed using the *vcalc* function, and a multivariate model was fitted using the *rma.mv* function, specifying the random-effects structure and fitting the model via restricted maximum likelihood estimation.

We assumed a constant within-study correlation of ρ = 0.30 between sampling errors, following prior influential studies, systematic reviews, and meta-analyses (e.g., 35, 75, 76). While the true correlation may vary across studies, this assumption allows for a principled modelling of within-study dependence and is further mitigated by the use of robust variance estimation to guard against model misspecification. In fact, since the variance-covariance matrix is only a rough approximation and the random-effects structure may not fully capture all dependencies in the underlying real outcomes/effects, a cluster-robust inference method - also known as ‘robust variance estimation’ - was applied to the model via the *clubSandwich* package (74). Tests of individual coefficients used a t-distribution, with degrees of freedom approximated using the Satterthwaite method. Omnibus tests were based on an F-distribution, with degrees of freedom approximated using an approximate Hotelling’s T-squared distribution (77–79).

To examine which coefficients might moderate the outcome, three mixed-effects meta-regression models were examined, each with multiple coefficients, as follows (73, 80):

1. Cognitive domains: set switching, planning/reasoning, memory, attention, working memory, verbal fluency, verbal semantic, abstraction, social cognition, and inhibition.
2. Control group typology: healthy controls, nonsexual offenders, sexual offenders against adults, internet offenders, child pornography users, and individuals with paraphilia or unspecified mental conditions.
3. Study group typology: individuals with (P+CSO) or without (CSO) a formal diagnosis of paedophilia.

For each model, we assessed the significance of both the overall model and individual coefficients, with and without the inclusion of the intercept (where appropriate). Coefficients were transformed into dummy coded variables by means of the *factor* function in R. Including the intercept allows comparisons against a reference category; while excluding it estimates effects for each category independently, so that coefficients represent average effect sizes for the corresponding category. This dual approach improves interpretability and ensures that findings are not dependent on arbitrary reference choices, providing a more comprehensive view of moderator effects.

Heterogeneity was evaluated using the restricted maximum likelihood estimator (81) and the Q-test for heterogeneity (82). Studentized residuals and Cook’s distances were employed to identify potential outliers and influential studies in the context of the present statistical model (80). On the one hand, studies with studentized residuals exceeding the $100\;\times {\left(1-\frac{0.05}{2k}\right)}^{th}$ percentile of a standard normal distribution were flagged as potential outliers using a two-sided Bonferroni correction. On the other hand, studies with a Cook’s distance greater than the median and six times the interquartile range of the Cook’s distance were considered influential, indicating that these studies had a disproportionate impact on the overall model fit.

Funnel plot asymmetry was assessed using two statistical tests: the rank correlation test (83) and the regression test (84), with the standard error of observed outcomes serving as the predictor variable. Outlying cases were evaluated based on their influence on the overall model. An outlier was considered inconsequential if it exerted minimal impact on the results. However, if removing a study led to substantial changes in the fitted model, the study was deemed influential (80). To identify influential studies, case-deletion diagnostics, known from linear regression (e.g., 85), were adapted to the meta-analysis by means of the *influence()* function, which provided leave-one-out diagnostics for each study, including: (1) externally standardised residuals, to detect unusual deviations; (2) difference in fits (DFFITS), to measure the influence of each study on the fitted values; (3) Cook’s distances, to assess the overall impact of a study on the model; (4) covariance ratios, to identify changes in model stability; (5) DFBETAS values, to evaluate the influence of individual observations on specific coefficients; (6) estimates of Tau², to measure between-study variance (heterogeneity) when removing each study; (7) heterogeneity test statistics, to assess changes in residual heterogeneity; (8) hat matrix diagonal elements, to evaluate leverage; (9) model weights, to determine the contribution of each study during model fitting.

Finally, the analyses were repeated with the study group split into two subgroups: individuals who committed sexual offenses against children with a formal diagnosis of paedophilia and those without, to examine differences linked to sexual preference versus offending behaviour.

### 2.2 Systematic review on acquired CSO

#### 2.2.1 Inclusion and exclusion criteria

Studies were included if they: (a) provided original reports of late onset CSO; (b) documented an organic condition temporally associated with the emergence of CSO.

Studies were excluded if they: (a) described the emergence of paedophilia without documenting CSO; (b) included patients with medical conditions who manifested CSO prior to the onset of the illness. Of note, the presence of paedophilia (i.e., attraction towards children upon which the individual has not acted) before the onset of the medical condition was not considered an exclusion criterion.

#### 2.2.2 Data selection and extraction

Cases of acquired CSO were identified through an existing systematic review (62) conducted in accordance with PRISMA guidelines (72), which was subsequently updated to include more recent studies. In particular, the original search string was run another time: ((pedophilia) OR (pedophilic behavio*) OR (child sex* offen*) OR (child molest*) OR (sex offen* against child*)) AND ((acquired) OR (*de novo*) OR (dementia) OR (brain lesion) OR (neurology*) OR (late onset)).

Two authors (CS, CC) independently extracted and screened the data, with random verification by a third author (SF). Extracted data included: neurological aetiology, brain localization, neurological symptoms, and cognitive impairments. Furthermore, based on the patient’s description, additional information was gathered regarding cognitive functioning, with particular attention to social cognition (specifically the construct of theory of mind, the ability to understand the social and moral disvalue of one’s actions, and the ability to discriminate right from wrong) and impulsive behaviour.

#### 2.2.3 Statistical analysis

Given the reliance on case reports, only descriptive statistics were computed. The percentage of patients presenting intact versus impaired cognitive functions was calculated across cases.

## 3 Results

### 3.1 Meta-analysis on developmental CSO

#### 3.1.1 Selected studies

The bibliographical search identified 163 entries. After duplicates removal, 148 records were screened. We excluded 70 articles as they did not meet the eligibility criteria, being: reviews (*n* = 7), meta-analyses (*n* = 5), case reports (*n* = 5), papers describing cases of acquired CSO (*n* = 2), studies presenting a new nonpharmacological treatment (*n* = 4), clinical trials (*n* = 5), hands-on (P+CSO/CSO) and hands-off (P-CSO) mixed study groups, or unrelated papers (*n* = 41). The remaining 78 records underwent full-text assessment, leading to the exclusion of 44 articles for the following reasons: (a) lack of cognitive or neuropsychological data (*n* = 31); (b) absence of a control group (*n* = 10); (c) insufficient statistical information to compute effect sizes (*n* = 3).

Ultimately, 34 articles were included in the meta-analysis, comprising 4093 subjects: 846 P+CSO, 1110 CSO, and 2137 controls (please see **Supplementary Table 1**). Control groups included healthy individuals (*n* = 966) and other subgroups such as: individuals with paraphilia (*n* = 56), nonsexual offenders (*n* = 306), online child offenders (*n* = 505), individuals with unspecified mental conditions (*n* = 15), adult sexual offenders against adults (*n* = 187), and child pornography users (*n* = 61).

**Figure 1:**
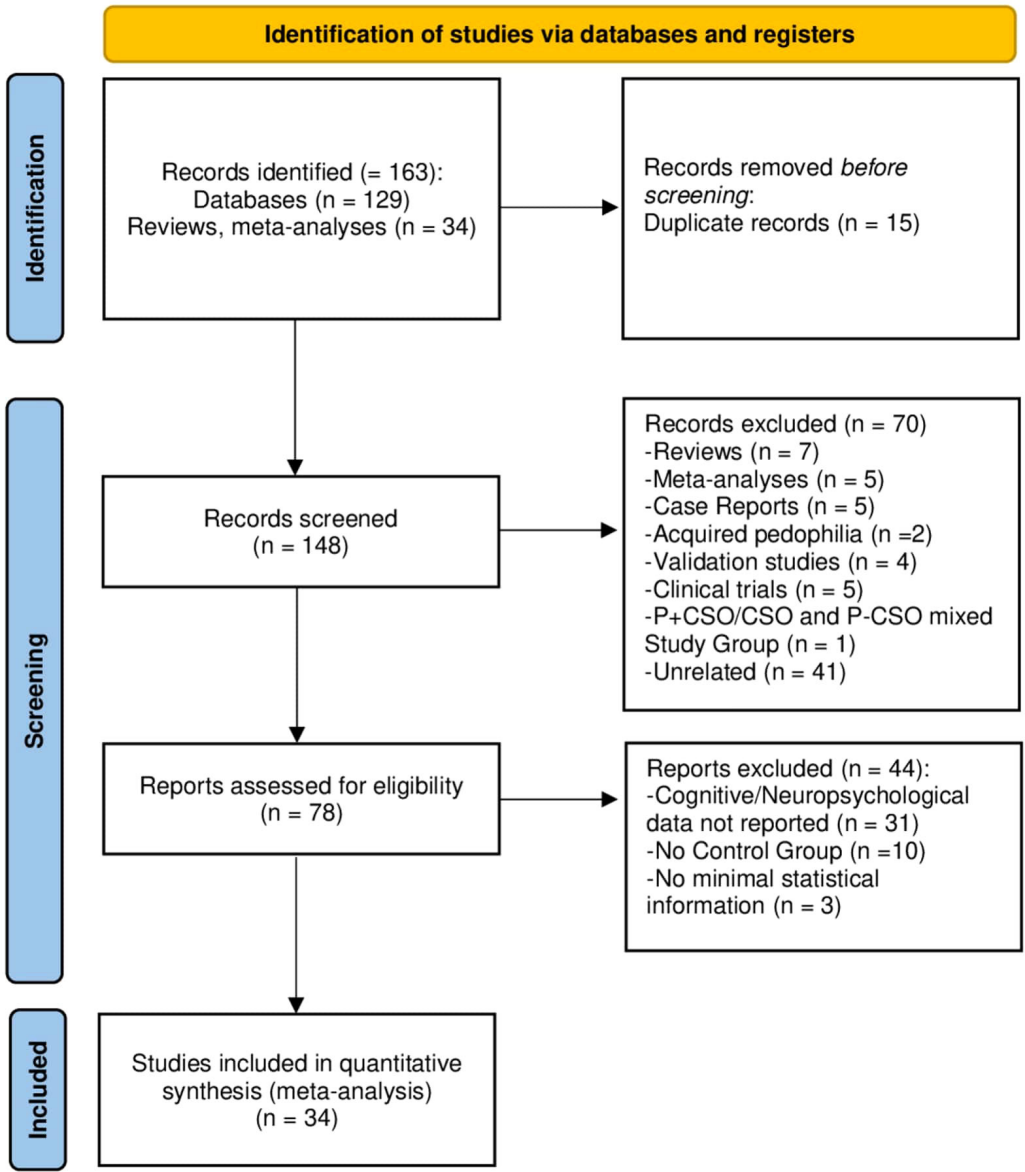
PRISMA flowchart illustrating the selection process of the present meta-analysis for developmental CSO.

**Figure 1** shows the PRISMA flowchart of the search strategy and the selection of the studies for the meta-analysis.

#### 3.1.2 Publication bias and influential effect sizes

**Figure 2:**
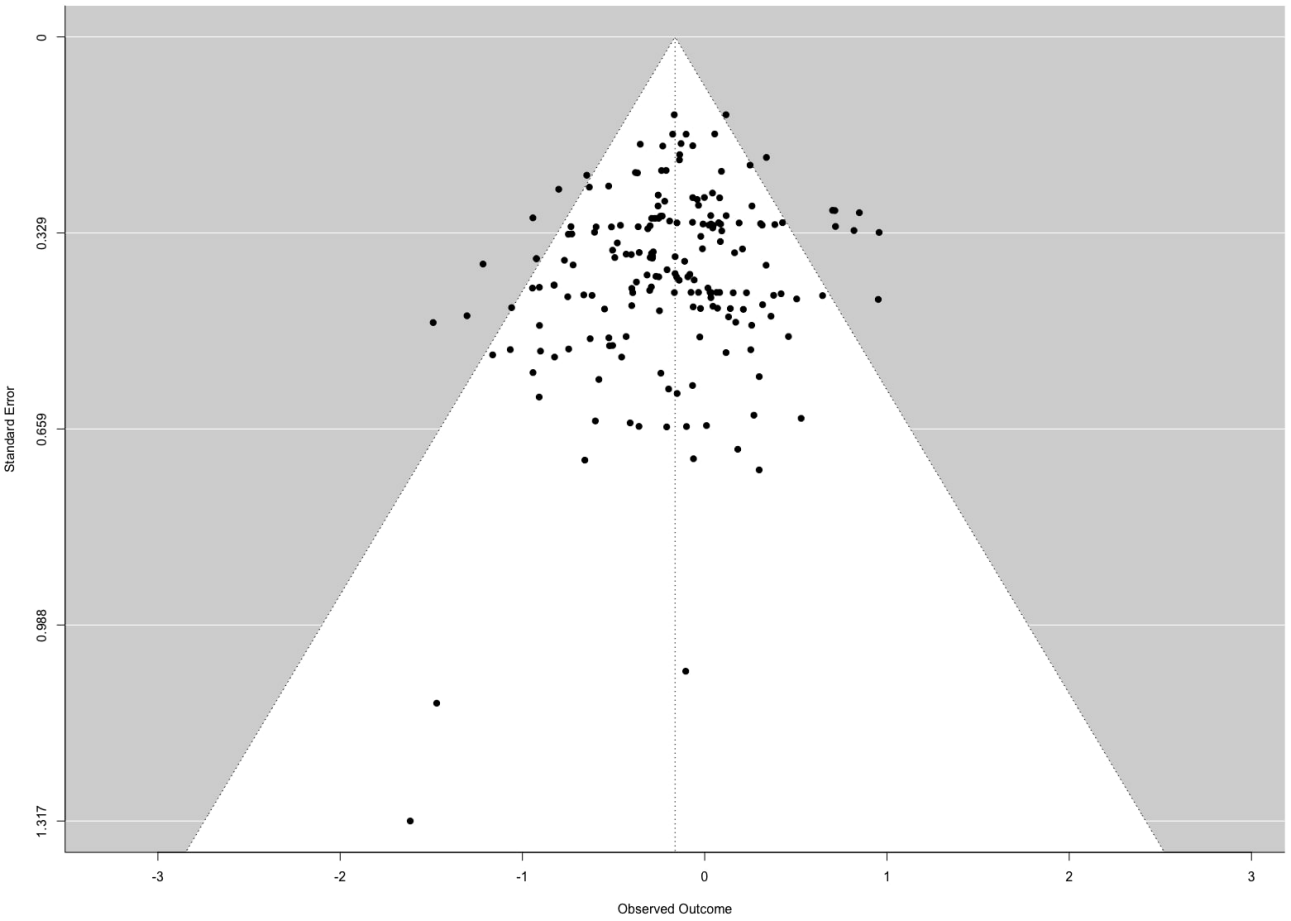
Funnel plot of the 34 studies included in the meta-analysis.

The 34 included studies accounted for 192 effect sizes. The Q-test revealed significant heterogeneity among true outcomes (*Q*(191) = 247.471, *p* = .004). Examination of studentized residuals revealed no values exceeding ±3.652, indicating no outliers within the context of the model. However, Cook’s distances flagged two effect sizes as overly influential. Case-deletion diagnostics (**Supplementary Figure 1**) confirmed that the absolute DFFITS values for these two effect sizes exceeded the threshold of $3\times \sqrt{\frac{p}{k-p}}$ , where *p* represents the number of model coefficients and *k* the total number of observations/effect sizes. The removed effect sizes were: −1.115 (DFFITS: −0.215) from Herrero et al. (86), and −1.735 (DFFITS: −0.264) from Becerra-Garcia & Egan (87). After outlier removal, the final analysis included 190 effect sizes from 34 studies. Despite the removal of these outliers, heterogeneity among true outcomes remained significant (*Q*(189) = 224.649, *p* = .039). Examination of the studentized residuals indicated the absence of outliers in the context of this model. The regression test detected funnel plot asymmetry (*p* = .03) (**Figure 2**), while the rank correlation test did not (*p* = .010). Residual heterogeneity (*Q_E_*-test) was significant when study group typologies were included as coefficients, but it was not significant in the mixed-effects meta-regression models that accounted for cognitive domains or control group typologies. In summary, once cognitive domains and control group typologies were included as coefficients, the variability between studies was no longer statistically significant, suggesting these factors help explain the observed heterogeneity.

#### 3.1.3 Multivariate meta-analysis

Across all effect sizes, observed outcomes ranged from −1.615 to 0.957, with 67% of estimates being negative. The random-effects model fitted with a cluster-robust inference method yielded a pooled effect size of *μ* = −0.186 (95% CI: −0.296 to −0.077, *p* = .002), indicating that individuals in the study group achieved worse cognitive/neuropsychological performances compared to controls (see **Table 1**). To examine which factors contributed to this result, mixed-effects meta-regression analyses were conducted.

**Table 1:** Overall: Multivariate meta-analysis; Domain, Study group, Control group: Mixed-effects meta-regression models.

	Estimate (SE)	t	*df*	p	CI Lower bound	CI Upper bound	Significance code
Overall	-0.186 (0.052)	-3.558	20.33	.002	-0.296	-0.077	**
Domain							
Set Switching	-0.139 (0.072)	-1.975	9.99	.076	-0.297	0.017	
Planning/Reasoning	-0.060 (0.106)	-0.564	14.81	.580	-0.287	0.167	
Memory	-0.278 (0.076)	-3.648	8.68	.005	-0.452	-0.104	**
Attention	-0.073 (0.109)	-0.673	11.66	.513	-0.311	0.164	
Working Memory	-0.268 (0.119)	-2.247	8.90	.051	-0.539	0.002	
Verbal Fluency	-0.268 (0.089)	-3.018	8.22	.016	-0.472	-0.064	*
Verbal Semantic	-0.260 (0.115)	-2.259	5.75	.066	-0.545	0.024	
Abstraction	0.217 (0.494)	0.439	1.07	.732	-5.124	5.558	
Social Cognition	-0.113 (0.186)	-0.605	4.20	.575	-0.621	0.395	
Inhibition	-0.364 (0.069)	-5.267	10.98	.000	-0.516	-0.212	***
Study group							
CSO	-0.214 (0.079)	-2.693	12.71	.0187	-0.386	-0.042	*
P+CSO	-0.155 (0.053)	-2.930	11.39	.0132	-0.272	-0.039	*
Control group							
HC	-0.313 (0.079)	-3.962	15.98	.001	-0.481	-0.145	**
NSO	-0.084 (0.071)	-1.178	7.00	.277	-0.254	0.085	
ASO	-0.081 (0.142)	-0.570	2.15	.622	-0.656	0.493	
IO/CP	0.078 (0.079)	0.985	2.58	.407	-0.200	0.357	
Other	-0.145 (0.033)	-4.328	1.00	.144	-0.571	0.281	

Results based on cluster-robust inference (variance-covariance estimator: bias reduced linearization; t-test, degrees of freedom and confidence intervals: Satterthwaite approximation). Significance codes: ‘***’.001; ‘**’.01; ‘*’.05; ‘.’.1. CSO: Individuals who committed sexual offenses against children. P+CSO: Individuals who committed sexual offenses against children and received a diagnosis of pedophilia. HC: Healthy Controls. NSO: Individuals who committed nonsexual offenses. ASO: Individuals who committed sexual offenses against adults. IO/CP: Individuals who committed offenses through internet and who used child pornography. Other: individuals with a paraphilia not otherwise specified or with an unspecified mental condition.

#### 3.1.4 Mixed-effects meta-regression

Mixed-effects meta-regression identified significant effects inthree cognitive domains: memory (*t* = -3.648, *p* = .005), verbal fluency (*t* = −3.018, *p* = .016), and inhibition (*t* = -5.267, *p* = .000) (**Table 1**). However, the omnibus F-test evaluating the joint significance of all coefficients was not significant (F = 3.027, df1 = 10, df2 = 3.03, *p* = .194). In other words, while the study group showed significantly worse performance than controls in these specific domains, the combined set of domain-level coefficients did not explain a statistically significant amount of variance in cognitive performance.

Meta-regression by study group revealed significant effects for both groups: CSO (*t* = -2.693, *p* = .0187) and P+CSO (*t* = -2.930, *p* = .0132) (**Table 1**), indicating worse cognitive/neuropsychological performance in each subgroup relative to controls. The omnibus test for this model was also significant (F = 6.159, df1 = 2, df2 = 15.44, *p* = .011), suggesting that, collectively, the set of coefficients for the study subgroups accounted for a significant portion of the variance in performance. A direct comparison between the P+CSO and CSO subgroups (with CSO set as the intercept) revealed no significant difference (*t* = 0.681, *p* = .503) (**Table 2**).

**Table 2:** Mixed-effects meta-regression models with intercept.

	Estimate (SE)	t	*df*	p	CI Lower bound	CI Upper bound	Significance code
Study group							
Intercept	-0.214 (0.079)	-2.693	12.71	.018	-0.386	-0.042	*
P+CSO	0.058 (0.086)	0.681	19.82	.503	-0.120	0.238	
Control group							
Intercept	-0.313 (0.079)	-3.962	15.98	.001	-0.481	-0.145	**
NSO	0.229 (0.098)	2.317	11.98	.039	0.013	0.444	*
ASO	0.232 (0.152)	1.520	3.60	.210	-0.211	0.675	
IO/CP	0.392 (0.117)	3.353	3.33	.037	0.040	0.744	*
Other	0.168 (0.086)	1.960	1.25	.258	-0.517	0.854	

Significance codes: ‘***’.001; ‘**’.01; ‘*’.05; ‘.’.1. CSO: Individuals who committed sexual offenses against children. P+CSO: Individuals who committed sexual offenses against children and received a diagnosis of pedophilia. HC: Healthy Controls. NSO: Individuals who committed nonsexual offenses. ASO: Individuals who committed sexual offenses against adults. IO/CP: Individuals who committed offenses through the internet and who used child pornography. Other: individuals with a paraphilia not otherwise specified or with an unspecified mental condition.

Results based on cluster-robust inference (variance-covariance estimator: bias reduced linearization; t-test, degrees of freedom and confidence intervals: Satterthwaite approximation).

Analysis by control group typology showed a significant effect for the healthy controls’ coefficient (*t* = −3.962, *p* = .0011) (**Table 1**). The omnibus F-test was not significant (F = 2.918, df1 = 5, df2 = 2.82, *p* = .213). That is, the study group showed statistically significant worse cognitive/neuropsychological performances only when compared with healthy controls, and the combined set of coefficients for the control subgroups did not account for a statistically significant portion of the variance in cognitive performance. Direct comparisons between healthy controls, set as the intercept, and the other control subgroups revealed significantly different average effects for both individuals who committed nonsexual offenses (*t* = 2.317, *p* = .039) and individuals who committed offenses against children through the internet and used child pornography (*t* = 3.353, *p* = .037), compared to healthy controls (**Table 2**). No significant differences were found for other control groups, including sexual offenders against adults or individuals with paraphilias not otherwise specified (**Table 2**).

#### 3.1.5 Sensitivity analysis

To further explore whether a specific type of control group influenced the results of the meta-regression model with cognitive domains as coefficients, a meta-regression analysis was conducted for inhibition, memory, and verbal fluency, with control group as coefficients (**Table 3**; **Supplementary Figures 2, 3**, and **4**, respectively). Some coefficients were excluded due to insufficient effect sizes (**Table 3**). The meta-regression model was significant for healthy controls with respect to inhibition (t = -3.512, p = .010) and memory (t = -4.517, p = .011). However, omnibus tests for these models were nonsignificant (inhibition: F = 1.809, df1 = 4, df2 = 1.87, *p* = .396; memory: F = 3.599, df1 = 3, df2 = 2.06, *p* = .203). These mixed results complicate distinguishing true effects from potential Type I errors. Overall, individuals with developmental CSO (CSO and P+CSO) exhibited poorer performance in inhibition and memory performances compared to healthy controls.

**Table 3:** Mixed-effects meta-regression model with control group typologies as coefficients, restricted to inhibition, memory and verbal fluency.

Inhibition	Estimate (SE)	t	*df*	p	CI Lower bound	CI Upper bound	Significance code
Control group							
**HC**	**-0.206 (0.058)**	**-3.512**	**6.81**	**.010**	**-0.346**	**-0.066**	*
NSO	-0.474 (0.245)	-1.934	2.36	.173	-1.391	0.441	
ASO	-0.161 (0.115)	-1.399	2.39	.277	-0.589	0.265	
IO/CP	-0.214 (0.238)	-0.900	1.92	.466	-1.285	0.855	
**Memory**	Estimate (SE)	T	*df*	P	CI Lower bound	CI Upper bound	
Control group							
**HC**	**-0.450 (0.099)**	**-4.517**	**3.84**	**.011**	**-0.732**	**-0.169**	*
NSO	-0.221 (0.146)	-1.514	3.58	.212	-0.648	0.204	
ASO	-0.115 (0.119)	-0.971	1.35	.473	-0.955	0.723	
**Verbal Fluency**	Estimate (SE)	T	*df*	P	CI Lower bound	CI Upper bound	
Control group							
HC	-0.590 (0.223)	-2.644	2.3	.102	-14.42	0.260	
NSO	-0.239 (0.088)	-2.783	3.2	.063	-0.502	0.024	

Results based on cluster-robust inference (variance-covariance estimator: bias reduced linearization; t-test, degrees of freedom and confidence intervals: Satterthwaite approximation). Significance codes: ‘***’.001; ‘**’.01; ‘*’.05; ‘.’.1. CSO: Individuals who committed sexual offenses against children. P+CSO: Individuals who committed sexual offenses against children and received a diagnosis of pedophilia. HC: Healthy Controls. NSO: Individuals who committed nonsexual offenses. ASO: Individuals who committed sexual offenses against adults. IO/CP: Individuals who committed offenses through internet and who used child pornography.

#### 3.1.6 Multivariate meta-analysis and mixed-effects meta-regression with split study group

The random-effects model on the CSO group, fitted with a cluster-robust inference method, produced a pooled effect size of μ = −0.232 (95% CI: −0.405 to −0.060, *p* = .011), indicating significantly poorer cognitive/neuropsychological performance in the CSO group compared to controls (**Table 4**). Mixed-effects meta-regression revealed significant domain effects for memory (*t* = -5.601, *p* = .002), verbal fluency (*t* = −2.910, *p* = .031), and inhibition (*t* = -3.058, *p* = .015). As for the typology of control group, a significant effect was also observed for the healthy controls’ coefficient (*t* = -3.610, *p* = .003) (**Table 4**).

**Table 4:** Main analyses divided for study groups.

	Estimate (SE)	t	*df*	p	CI Lower bound	CI Upper bound	Significance code
*CSO - Overall*	-0.232 (0.080)	-2.887	14.21	.011	-0.405	-0.060	*
CSO - Domain							
Set Switching	-0.140 (0.091)	-1.546	7.45	.163	-0.353	0.072	
Planning/Reasoning	-0.074 (0.130)	-0.570	9.17	.582	-0.369	0.220	
Memory	-0.430 (0.076)	-5.601	4.99	.002	-0.627	-0.232	**
Attention	-0.158 (0.210)	-0.750	5.72	.482	-0.680	0.363	
Working Memory	-0.444 (0.190)	-2.334	5.20	.064	-0.928	0.039	
Verbal Fluency	-0.311 (0.106)	-2.910	5.20	.031	-0.583	-0.039	*
Verbal Semantic	-0.259 (0.141)	-1.833	3.14	.160	-0.698	0.179	
Abstraction^§^	0.414 (0.101)	4.095	4.17	.013	0.137	0.690	*
Social Cognition	-0.256 (0.297)	-0.863	3.06	.451	-1.197	0.678	
Inhibition	-0.339 (0.111)	-3.058	8.25	.015	-0.594	-0.084	*
CSO - Control group							
HC	-0.400 (0.110)	-3.610	12.00	.003	-0.642	-0.145	**
NSO	-0.110 (0.078)	-1.415	7.29	.198	-0.293	0.085	
ASO	0.082 (0.111)	0.734	2.92	.517	-0.279	0.493	
*P+CSO - Overall*	-0.153 (0.050)	-3.021	10.15	.012	-0.266	-0.040	*
P+CSO - Domain							
Set Switching	-0.213 (0.074)	-2.881	4.23	.042	-0.414	-0.012	*
Planning/Reasoning	-0.038 (0.155)	-0.250	6.50	.809	-0.411	0.333	
Memory	-0.152 (0.073)	-2.058	3.27	.124	-0.376	0.072	
Attention	-0.020 (0.088)	-0.231	5.71	.824	-0.240	0.199	
Working Memory	-0.161 (0.125)	-1.290	4.16	.263	-0.503	0.180	
Verbal Fluency	-0.254 (0.128)	-1.988	3.57	.126	-0.627	0.118	
Verbal Semantic	-0.278 (0.096)	-2.887	2.66	.072	-0.607	0.051	
Abstraction	0.115 (0.596)	0.193	1.06	.876	-6.484	6.715	
Social Cognition	-0.014 (0.219)	-0.067	1.89	.952	-1.015	0.986	
Inhibition	-0.399 (0.130)	-3.060	7.57	.016	-0.703	-0.095	*
P+CSO - Control group							
HC	-0.225 (0.074)	-3.016	9.37	.013	-0.393	-0.057	*
NSO	0.051 (0.090)	0.571	1.00	.669	-1.094	1.197	
ASO	-0.218 (0.016)	-13.295	1.00	.047	-0.427	-0.009	*
IO/CP	0.117 (0.087)	1.346	1.61	.336	-0.360	0.596	
Other	-0.143 (0.032)	-4.440	1.00	.141	-0.553	0.266	

Results based on cluster-robust inference (variance-covariance estimator: bias reduced linearization; t-test, degrees of freedom and confidence intervals: Satterthwaite approximation). Significance codes: ‘***’.001; ‘**’.01; ‘*’.05; ‘.’.1. CSO: Individuals who committed sexual offenses against children. P+CSO: Individuals who committed sexual offenses against children and received a diagnosis of pedophilia. HC: Healthy Controls. NSO: Individuals who committed nonsexual offenses. ASO: Individuals who committed sexual offenses against adults. IO/CP: Individuals who committed offenses through internet and who used child pornography. Other: individuals with a paraphilia not otherwise specified or with an unspecified mental condition.

^§^Only one study contributed to the abstraction domain for the analysis restricted to the CSO study group, therefore the results should not be considered reliable.

Overall: Multivariate meta-analysis; Domain, Control group: Mixed-effects meta-regression models.

For the P+CSO group, the pooled effect size was μ = −0.153 (95% CI: −0.266 to −0.040, *p* = .012), again reflecting significantly worse cognitive performance compared to controls (**Table 4**). Mixed-effects meta-regression revealed domain-specific effects for set switching (*t* = -2.881, *p* = .042), and inhibition (*t* = -3.060, *p* = .016). Regarding control group typology, significant effects were identified for healthy controls’ coefficient (*t* = -3.016, *p* = .013), and for individuals who committed sexual offenses against adults (*t* = -13.295, *p* = .047) (**Table 4**).

### 3.2 Systematic review on acquired CSO

**Figure 3:**
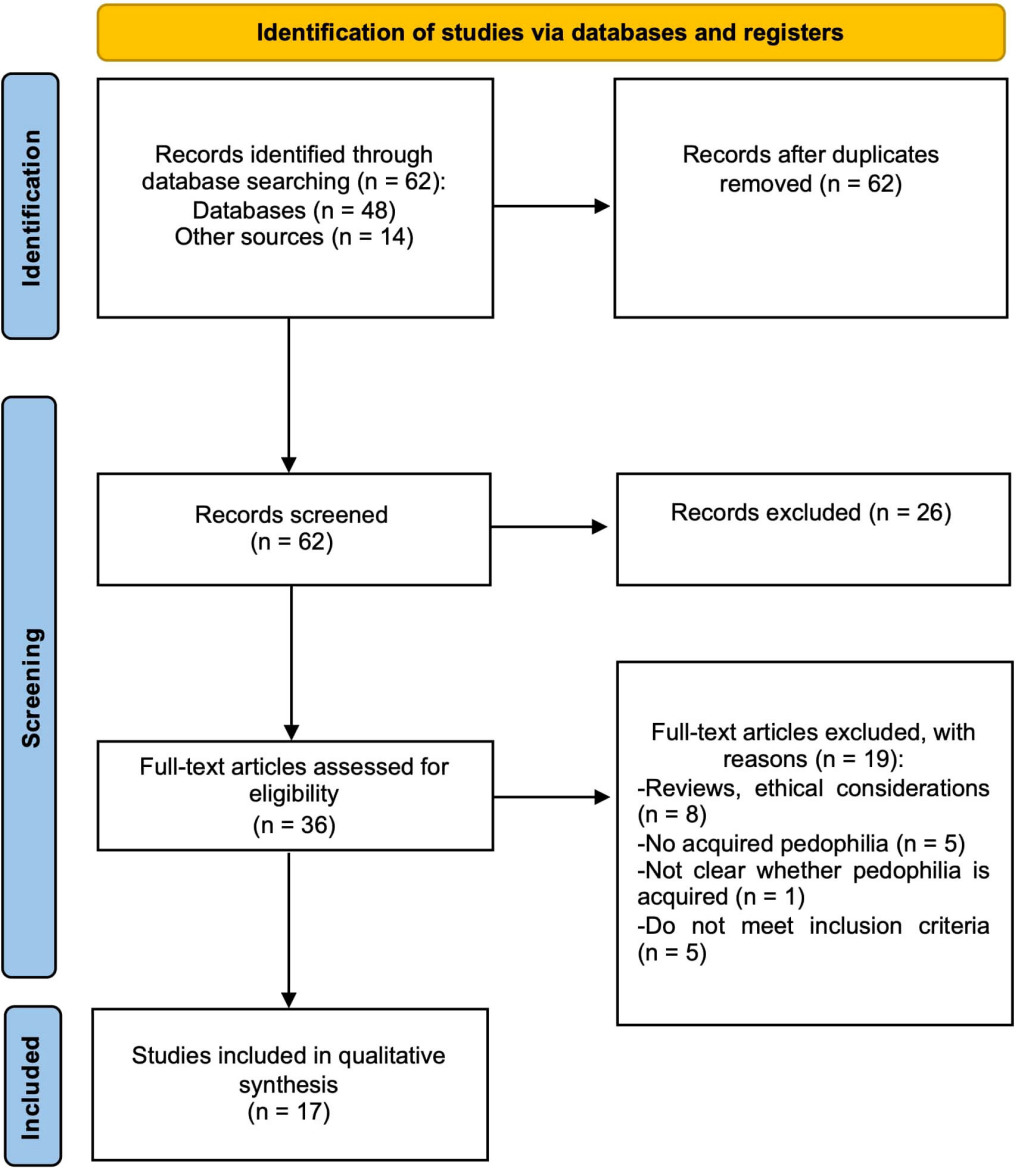
PRISMA flowchart illustrating the selection process of the systematic review on acquired CSO.

A total of 21 papers were identified through the literature search, describing 26 cases of late-onset CSO. The full-text analysis based on the eligibility criteria led to the inclusion of 21 cases from 17 papers (**Figure 3**). A summary of the brain pathologies and the corresponding anatomical localisation of lesions for each case of acquired CSO included in the systematic review is provided in **Supplementary Table S2**.

#### 3.2.1 Excluded studies

Several case reports were excluded based on the following specific criteria:

• **Lack of a neurological condition:**

Prahlada Rao et al. (88): the patient exhibited cognitive impairments, but no underlying neurological condition was identified as the MRI results were normal.

Regestein and Reich (89): patients 2, 3, and 4 were excluded due to the absence of a confirmed neurological condition.

• **Absence of child sexual offenses:**

Alnemari et al. (57): the patient displayed increased sexual interest in children following a traumatic brain injury (left basal frontal and bilateral temporal contusions), but did not commit sexual offenses against children.

• **Offenses predating the neurological condition:**

Prado et al. (25): a patient with frontotemporal dementia was excluded after it was discovered that sexually inappropriate behaviours toward his daughter occurred years before the onset of the neurological condition.

Mendez et al. (65) and Mendez (58): both cases were excluded because the individuals committed child sexual offenses prior to the onset of their neurological conditions.

#### 3.2.2 Neuropsychological results

**Table 5** provides an overview of the neuropsychological profiles of patients with acquired CSO, focusing on the same cognitive domains assessed for developmental CSO. While most studies reported that formal neuropsychological evaluations had been conducted, specific details such as test scores and the names of the assessment techniques employed were frequently absent. However, the available descriptions of patients’ daily-life challenges, offenses, and *modus operandi* allowed a comprehensive evaluation of critical cognitive functions, particularly social cognition and impulse control (see **Supplementary Table 2** for details). Interestingly, all but two patients displayed neurological symptoms alongside cognitive impairment, consistent with the neurological nature of acquired CSO.

**Table 5:** Neuropsychological profile of patients with acquired CSO.

Reference	Inhibition	Social cognition	Task switching	Executive functions	Abstraction	Memory	Working memory	Attention	Verbal fluency	Semantic	Other
Lesniak et al., (90)	Impaired	n/a	n/a	n/a	n/a	n/a	n/a	n/a	n/a	n/a	Low IQ*
Regestein & Reich (89) case 1	Impaired	Impaired	n/a	n/a	n/a	n/a	n/a	Impaired*	n/a	n/a	Impaired WCST*
Miller et al. (24)	Impaired	Impaired	n/a	n/a	n/a	n/a	n/a	n/a	n/a	n/a	n/a
Ortego et al. (91)	n/a	n/a	n/a	n/a	n/a	n/a	n/a	n/a	n/a	n/a	IQ=78
Dimitrov et al (92)	Impaired*	Impaired*	Borderline*	Spared*	Spared*	Impaired*	Spared*	Impaired*	Spared*	n/a	n/a
Frohman et al (37)	Spared* Impaired	Spared	n/a	n/a	n/a	Impaired*	n/a	Spared*	Spared*	Spared*	Spared WCST Normal IQ
Burns & Swerdlow (13)	Spared* Impaired	Spared	Spared*	n/a	n/a	Spared*	Spared*	n/a	Impaired*	n/a	MMSE impaired, Constructional apraxia, agraphia
Solla et al. (18)	Impaired	Spared	n/a	n/a	n/a	n/a	n/a	n/a	n/a	n/a	n/a
Devinsky et al (28)	Impaired	Spared	n/a	n/a	n/a	n/a	n/a	n/a	n/a	n/a	n/a
Mendez & Shapira (19), case 2	Impaired	Impaired	n/a	Impaired*	n/a	Impaired*	n/a	Spared*	Impaired*	n/a	Spared visuo-spatial construction
Mendez & Shapira (19), case 3	Impaired	n/a	n/a	Impaired*	n/a	Impaired*	n/a	Spared*	Spared*	n/a	Impaired visuospatial construction
Mendez & Shapira (19), case 4	Impaired	n/a	n/a	Impaired*	n/a	Impaired*	n/a	Impaired*	Impaired*	n/a	Perseverations; Impaired visuospatial construction
Mendez & Shapira (19), case 5	Impaired	Spared	n/a	Spared*	n/a	Spared*	n/a	Spared*	Spared*	n/a	Spared apraxia
Mendez & Shapira (19), case 6	Impaired	n/a	n/a	Impaired*	n/a	Spared*	n/a	n/a	Impaired*	Spared*	n/a
Mendez & Shapira (19), case 7	Impaired	Spared	n/a	Spared*	n/a	n/a	n/a	Spared*	Spared*	Spared*	Spared calculations
Rainero et al (93)	n/a	Impaired*	n/a	n/a	n/a	n/a	n/a	n/a	n/a	n/a	MMSE 28.5/30 Selective impairment in frontal functions
Fumagalli et al (38)	Impaired*	Impaired*	n/a	n/a	n/a	n/a	n/a	n/a	n/a	n/a	n/a
Gilbert & Vranic; Gilbert et al. (29, 94)	n/a	n/a	n/a	n/a	n/a	n/a	n/a	n/a	n/a	n/a	n/a
Sartori et al.; Scarpazza, Pellegrini et al. (26, 30)	Impaired*	Impaired*	Spared*	Spared*	Spared *	n/a	n/a	Spared*	Impaired*	n/a	Simultanagnosia
Scarpazza et al., (23), case 1	Impaired*	Impaired*	n/a	Impaired*	n/a	Spared*	n/a	Impaired*	n/a	n/a	n/a
Scarpazza et al., (23), case 2	Impaired*	unclear	n/a	Impaired*	n/a	n/a	n/a	Impaired*	n/a	n/a	Constructional apraxia

Asterisk (*) denotes data from formal neuropsychological assessment. Its absence denotes that the data was deduced by the clinical description of the patient provided in the paper. n/a denotes that the information is not available. IQ, Intelligence quotient; WCST, Wisconsin Card Sorting Test; MMSE, Mini Mental State Examination.

##### Key findings

- Social cognition: information on the ability to understand the social and moral disvalue of sexual offenses was available for 16 out of 21 cases (76%). Of these, 62.5% (10/16) demonstrated impaired understanding of the wrongfulness of their actions, while 37.5% (6/16) retained this ability.
- Impulse control: sufficient data on impulse control was provided in 19 of the 21 cases (90.5%), with all individuals (100%) exhibiting significant impulsivity at the time of the crime. This impulsive behaviour was also observed in their everyday lives, further supporting its pathological nature.
- Cognitive domains: impairments were frequently noted across a range of cognitive domains. Executive functions were assessed in 13 patients, with 61.5% (8/13) showing deficits. Verbal fluency, evaluated in 10 cases, was impaired in 60% (6/10). Memory, tested in 9 patients, was defective in 55.5% (5/9). Lastly, attention abilities, assessed in 11 patients, were found to be impaired in 45.5% (5/11).

## 4 Discussion

In the current study, we examined the cognitive profile of individuals who committed sexual offenses against children, aiming to frame their behaviour in the context of its neuropsychological underpinnings. To this end, we conducted a meta-analysis on 34 studies, including a total of 846 individuals with P+CSO, 1110 individuals with CSO, and 2137 controls, alongside a systematic review of 21 cases of acquired CSO.

### 4.1 Summary of the results

The meta-analytical approach revealed that individuals with developmental CSO tend to exhibit a cognitive profile characterised by impairments in inhibition, memory, and verbal fluency compared to healthy controls. The deficits observed were of medium effect size, according to Cohen’s thresholds (95, 96). Given the complexity of the statistical models and the number of analytical decisions involved, readers are advised to interpret the present results with caution, as they may be influenced by model specifications and underlying assumptions.

Importantly, when individuals with developmental CSO were analysed separately, based on whether they had a formal diagnosis of paedophilia, individuals without a formal diagnosis of paedophilia (CSO) demonstrated impairments in memory, verbal fluency, and inhibition compared to healthy controls, whereas individuals with a formal diagnosis of paedophilia (P+CSO) showed deficits in set-switching and inhibition compared to both healthy controls and individuals who committed sexual offenses against adults. Of note, these cognitive functions are closely linked to frontal lobe activity (97, 98) and are typically categorised as executive functions. A plausible explanation for these findings lies in the methodological approaches of the included studies. While the CSO group was defined as lacking a formal diagnosis of paedophilia, a detailed examination of the studies suggests that many individuals in this group likely exhibited undiagnosed paedophilic tendencies. For instance, Bartels et al. (99) reported increased sexual interest in children among subjects with CSO compared to controls. Moreover, Turner et al. (100) found that CSO individuals rated images of children as significantly more sexually arousing than controls. Finally, Veneziano et al. (101) highlighted that CSO individuals included in their study exhibited known risks factors for paedophilic disorder. These observations suggest that the CSO group likely included a substantial proportion of individuals with undiagnosed paedophilic disorder, which could have influenced the results.

This phenomenon of underdiagnosis is well documented in forensic settings, where paedophilic disorder is frequently not formally identified, even when clinical indicators are present. Several factors may contribute to this trend. First, the pervasive stigma surrounding the diagnosis may lead to reluctance among clinicians to assign it explicitly. Second, diagnostic assessments are often limited by a lack of access to specialised tools and a reliance on self-report measures, which may be distorted by exaggeration, minimisation, or intentional misrepresentation (11). Third, implicit biases may influence evaluators and legal professionals, who may be more inclined to interpret child sexual offending as deliberate and volitional rather than symptomatic of underlying psychopathology, particularly in light of the moral and emotional weight these offences carry. Moreover, in some judicial contexts, there may be institutional resistance to accepting paedophilic disorder as a mitigating factor. Concerns may arise that recognising a psychiatric diagnosis could be seen as diminishing individual responsibility, thus provoking public outrage. However, it is important to emphasise that the attribution of a psychiatric diagnosis does not automatically imply insanity; rather, a separate determination must be made regarding the causal relationship between the mental disorder and the offence, as usually required for assessments of criminal responsibility (e.g., Italian Penal Code; American Model Penal Code §4.01). These systemic and contextual influences likely contribute to the underrecognition of paedophilic disorder in forensic populations, and they should be carefully considered when interpreting the clinical composition and cognitive findings associated with the CSO group.

The systematic review of individuals with acquired CSO, though lacking the quantitative synthesis that enhances the strength of evidence in meta-analyses, provided additional insights. All individuals with acquired CSO manifested impulsivity, and over half showed concomitant deficits in social cognition, specifically in theory of mind abilities and moral reasoning. These deficits were evident in formal neuropsychological assessments and corroborated by reports from relatives about their everyday behaviours. These findings are particularly noteworthy, as the cognitive impairments observed in acquired CSO are not only measurable but also clinically significant at the individual level, underscoring the profound impact of neurological conditions on behaviour and moral judgment.

### 4.2 Implications for the debate on cognitive functioning

#### 4.2.1 Developmental CSO

Our findings on developmental CSO quantitatively support and extend prior research (43, 102). Consistent with a recent systematic review (102), we observed that individuals with CSO exhibit impaired executive functions, particularly in inhibition and set-switching, while abstraction and planning abilities remain intact. Additionally, our results refine the conclusions of a prior review by Dillien et al. (68), showing that while CSO and P+CSO share overlapping neuropsychological deficits, there are some distinctions. For instance, our meta-analysis clarifies that verbal fluency deficits are specific to CSO and not present in P+CSO when compared to healthy controls. However, unlike prior studies, we did not find evidence of social cognitive deficits in CSO individuals (68). This discrepancy may highlight methodological variations across studies or suggest a more nuanced relationship between social cognition and sexual offending behaviour.

Our results also corroborate previous findings suggesting that executive dysfunction is related to the offending behaviour rather than paedophilic tendencies (42). The shared inhibitory deficits observed in both CSO and P+CSO groups support the motivation-facilitation model of sexual offending proposed by Seto (103), which posits that sexual offenses occur when self-regulation mechanisms fail to suppress inappropriate sexual desires, independently of the target of said desires. Importantly, individuals with paedophilia who do not commit offenses generally do not exhibit inhibitory control deficits (41, 42, 100, 102, 104, 105), although exceptions exist (106).

Despite a failure in the ability to regulate one’s behaviour has been identified as an important predictor for sexual recidivism (107), it remains uncertain whether this impairment in behavioural control is specific to sexual offences against children or indicative of broader antisocial tendencies (108–110). Furthermore, it is still unclear if the findings of diminished cognitive control at formal testing in CSO and P+CSO could be translated to complex decision-making processes in real-life scenarios (42). In our opinion, this behaviour cannot be interpreted as an inability to restrain preponderant action, because sexual offences committed by individuals with developmental CSO cannot be considered impulsive (63).

An interesting hypothesis is that inhibitory deficits observed in P+CSO might be explained by an increased effort required to redirect attention from dominant tendencies (105). This hypothesis is supported by findings of increased Stroop task interference in P+CSO individuals, which correlates with heightened conflict-related activity in the superior parietal lobe and precentral gyrus (105). The authors of this study suggest that potential difficulties in attention reallocation may account for poor impulse control and moderate the risk of committing CSO (105). This hypothesis aligns with our finding that inhibition deficits often co-occur with set-switching impairments in P+CSO individuals. Additional studies are needed to further explore this hypothesis and to test whether this pattern extends to CSO individuals without a formal paedophilia diagnosis.

#### 4.2.2 Acquired CSO

Our results on acquired CSO are consistent with prior research highlighting disinhibition as a hallmark of this condition (27), with hypersexuality often being a behavioural manifestation. However, our study extends previous findings by showing that over half of the individuals with acquired CSO also present deficits in social cognition and moral reasoning. These deficits align with the neurophenomenological model of sexual arousal proposed by Stoléru and colleagues (111), which posits the existence of three main components contributing to sexual arousal: inhibitory control, cognitive evaluation, and autonomic/endocrine processes. More specifically, our findings support the idea that both the inhibitory component (i.e., the ability to withhold the preponderant action) and the cognitive component (i.e., the ability to evaluate one’s own behaviour) are impaired in individuals with acquired CSO. For instance, individuals with acquired CSO often exhibit a sudden breakdown in socially appropriate behaviour, as emerged from formal neuropsychological evaluations and corroborated by reports from close relatives (**Supplementary Table 2**). This may include an inability to engage in proficient social interactions and a failure to adhere to ethical, social, and legal norms. Combined with behavioural disinhibition, these features help contextualize their criminal *modus operandi*. We propose that disinhibition alone may not be sufficient to explain CSO, because individuals who are disinhibited but still recognize the moral and legal wrongfulness of offending against children are less likely to seek opportunities to offend and are more likely to pursue therapeutic help. This concept aligns with the *actio libera in causa* principle (112), which suggests that while an individual may not have full control over their actions during the offense due to disinhibition, they still retain the ability to control the conditions that lead to the offense. According to this hypothesis, a significant proportion of men with developmental paedophilia, who do not typically exhibit impaired social cognition, have never committed sexual offenses and search for help (6, 7, 11). In contrast, when disinhibition is coupled with social cognition deficits, as seen in acquired CSO, individuals are less likely to recognize the wrongfulness of their impulses and thus are more likely to act on them. These findings suggest that acquired CSO is not merely the result of an isolated inhibitory dysfunction, but likely stems from a combined dysfunction in both inhibitory control and social cognition, as proposed in the neurophenomenological model of sexual arousal (111).

Importantly, the differences in the cognitive profile between developmental and acquired CSO identified in the current meta-analysis and systematic review, along with the established differences in the neural bases (59), caution against using acquired CSO as a model to investigate the potential neurobiological basis of developmental CSO, aligning with Joyal’s (27) earlier recommendations.

### 4.3 Implications for forensic practice

The cognitive profiles of individuals with developmental and acquired CSO offer insights into their distinct *modi operandi*, which reflect differences in underlying neuropsychological functioning.

#### 4.3.1 Developmental CSO

The *modus operandi* of individuals with developmental CSO is described in the literature as compulsive rather than impulsive (63). Offenses are often premeditated (63, 66, 67, 113), occurring in private settings and without witnesses, with offenders employing strategies to enforce the victim’s silence (63–65). This level of behavioural control suggests that their inhibitory abilities, while impaired according to formal cognitive evaluations, are sufficient to allow them to delay and structure their actions until conditions are favourable for offending. This apparent discrepancy between experimental findings of inhibitory deficits and observed behaviours in real-life settings could have several possible explanations. First, most of the studies provide group-level data, which may not translate uniformly to individual cases. Second, the effect sizes reported are generally small to moderate. For instance, in the case of inhibition, reaction times between groups usually differ by a few milliseconds. It is therefore very difficult to state that these results obtained in laboratory settings have clinical relevance. Third, developmental P+CSO has high comorbidity with other psychiatric disorders, particularly personality disorders (64, 114–117). This complicates the attribution of neuropsychological impairments to paedophilia alone. Fourth, it remains unclear whether inhibitory deficits observed in formal evaluations are stimulus-specific (e.g., triggered by child-related stimuli) or generalize to neutral contexts. Notably, individuals with developmental CSO have not been described as disinhibited in their daily lives.

#### 4.3.2 Acquired CSO

Different is the *modus operandi* of individuals with acquired CSO, which has been described as impulsive and disorganized (62). These individuals do not plan sexual offenses; rather, they act on an urge, and they do not try to mask their behaviour, which may occur in public places and in front of witnesses (23, 26). This *modus operandi* suggests that their inhibitory abilities are severely impaired, as they are unable to refrain from offending even in highly inappropriate or risky situations. Additionally, the lack of effort to conceal their actions suggests an impairment in understanding the moral and legal wrongfulness of their behaviour. This aligns with our findings, which revealed impaired inhibitory abilities in all (100%) individuals with acquired CSO and deficits in social cognition in 62.5% of cases. It is also worth noting that 61.5% of patients also manifested a general deficit in executive functions. These deficits in acquired CSO are not confined to formal neuropsychological evaluations but are also evident in daily life, as reported by relatives and caregivers. This provides robust evidence of their clinical relevance and highlights the profound impact of these impairments on behaviour.

Importantly, the identification of a brain lesion in these individuals should not be interpreted as sufficient, in itself, to explain or excuse sexually offending behaviour. In accordance with recommendations of the international consensus conference on acquired paedophilia (118) and established guidelines on the forensic use of neuroimaging (119), the presence of a neurological abnormality must be interpreted within the broader context of the individual’s cognitive and behavioural profile. Criminal responsibility and clinical risk assessments should be based primarily on demonstrable impairments in mental functioning - such as deficits in inhibition and/or moral reasoning - and on a clear causal relationship between these impairments and the offence. In this sense, neuroimaging findings serve as supportive evidence, but do not replace the need for thorough neuropsychological evaluation.

### 4.4 Summary of the two profiles

Developmental CSO often occurs in individuals with paedophilia or in those without any formal diagnosis. Brain alterations in developmental CSO, while present, are not macroscopically visible. These changes can only be detected through advanced brain imaging analyses (e.g., 15, 45, 120, 121) and are spatially heterogenous, as findings from different studies do not show convergence on specific brain regions and/or networks (23). From a cognitive perspective, our study identified deficits in inhibition, memory, and verbal fluency among individuals with developmental CSO. However, these cognitive impairments do not align with specific brain alterations, nor is there evidence that they significantly impact daily functioning or the *modus operandi*, which cannot be described as impulsive (63–65). Further research is needed to clarify the extent to which these cognitive deficits influence real-world behaviour.

In contrast, acquired CSO arises as a symptom of a neurological condition (13, 62, 91, 92). Neuroimaging consistently reveals visible lesions, which, despite being spatially heterogeneous, are linked to a disrupted network involving the orbitofrontal cortex and posterior midline structures, such as the posterior cingulate cortex and praecuneus (23). The functional characterization of these regions suggests that the orbitofrontal cortex supports impulse control, while the posterior midline structures are crucial for social cognition. Our findings revealed that impulse control deficits were present in all (100%) patients with acquired CSO, while deficits in social cognition were evident in 62.5%. This reflects a robust anatomo-clinical correspondence between brain alterations and cognitive impairments (122). Unlike developmental CSO, these impairments are apparent in daily life and significantly influence the modus operandi, which is characterized by impulsive, disorganized behaviour and a lack of awareness of the moral, social, and legal implications of offending (62). Acquired CSO is also associated with broader cognitive deficits, including impairments in attention, memory, verbal fluency, and executive functioning, as well as the presence of neurological symptoms that serve as “red flags” for the underlying organic condition (118).

### 4.5 Limitations and future directions

This study is not free from drawbacks. The primary limitation lies in the different methods applied to draw conclusions regarding the two forms of CSO. Studies on developmental CSO typically report group-level data, offering limited detail about individual cases. Conversely, research on acquired CSO consists exclusively of detailed single-case descriptions due to the rarity of the condition. Consequently, applying the same analytical approach to both groups was not feasible. A second limitation is related to the variability in how neuropsychological performance, neural bases, and *modus operandi* were studied. For developmental CSO, these aspects were studied across multiple individuals; therefore, we do not know the exact dynamic of the offense nor the neural dysfunction of the patients from the studies included in the current meta-analysis. In contrast, for acquired CSO, all relevant data (cognitive impairments, neural bases, and behavioural patterns) were derived from the same individuals, allowing for a more integrated analysis.

The present study also offers several important directions for future research. First, our findings highlight the need for studies that directly compare developmental and acquired CSO using comprehensive neuropsychological batteries, enabling more precise mapping of cognitive profiles across subtypes. Moreover, future research should investigate whether, and to what extent, specific cognitive deficits predict behavioural patterns, response to treatment, or risk of recidivism. Longitudinal studies would also be valuable to determine whether these neuropsychological impairments remain stable over time or are modifiable through intervention, and to identify which types of treatment are most effective for each subgroup.

Given the differences in aetiology between developmental and acquired CSO, we should expect differential responses to treatment. For instance, individuals with acquired paedophilia may benefit from interventions targeting the underlying neurological condition *–* such as tumour resection, management of neurodegenerative disease, or targeted rehabilitation for traumatic brain injury *–* which might lead to a significant reduction or even resolution of paedophilic behaviour. However, when medical treatment alone is insufficient to fully mitigate risk, particularly in the presence of persistent deficits in impulse control or social cognition, optimal management should involve a multidisciplinary approach. This may include ongoing medical care, neuropsychological rehabilitation, behavioural strategies, and structured supervision. Risk assessment protocols for this population should be tailored to account for neurological and cognitive contributors to risk, including potential relapse or progression of the underlying condition, disinhibition, diminished moral reasoning, and impaired insight. Such an approach is essential to ensuring both public safety and ethically grounded, individualised therapeutic intervention.

The management of developmental CSO, by contrast, presents substantial challenges. Unlike acquired forms, where treating the neurological condition can sometimes eliminate paedophilic behaviour, no consistently effective large-scale intervention currently exists for this condition (123). Low compliance with available therapeutic programmes further complicates prevention and risk management efforts (6). As a result, individuals with developmental CSO may present a higher risk of recidivism compared to those with acquired, where targeted medical intervention can directly address the underlying cause of the behaviour (124). Given that sexual interest in children tends to remain stable over time in developmental cases, long-term structured intervention and monitoring are essential to mitigate risk (125). These challenges underscore the pressing need for research focused on developing and validating more effective, ethically sound, and scalable interventions for developmental CSO, as well as refining risk assessment tools to better support clinical and forensic decision-making in this population.

### 4.6 Conclusions

This study provides support for the hypothesis that developmental and acquired CSO are associated with a distinct cognitive profile. Both groups exhibit deficits in inhibitory control, but social cognition impairments are present only in acquired CSO. Within developmental CSO, CSO and P+CSO individuals share similar cognitive profiles, suggesting that the CSO group likely includes individuals with undiagnosed paedophilia.

The results highlighted in the current paper have important implications regarding the appropriate classification of the CSO type and the respective potential therapeutic interventions. Regarding the classification, this study shows that impairments in social cognition might suggest the presence of an acquired origin of CSO. In respect of the possible interventions, we can speculate that individuals with acquired CSO may benefit from forms of cognitive rehabilitation focused on social cognition, whereas those with developmental CSO might require interventions targeting inhibitory control, potentially combined with pharmacological treatments.

Given the different prognoses of developmental and acquired CSO and the consequences of misidentification, accurately identifying the type of CSO is of critical relevance. A recent international consensus conference (118) suggested that a case-by-case analysis should always be warranted. Particularly, when impulsivity is noted in the *modus operandi*, a comprehensive neuropsychological evaluation – including, when appropriate, neuroimaging – should be conducted (21, 30, 126). Such investigation should include neuroimaging and an in-depth formal neuropsychological evaluation, mainly targeted on impulse inhibition and social cognition abilities. This study’s findings further support the consensus conference’s conclusions (118), emphasising that while developmental and acquired CSO share inhibitory deficits (evident in formal cognitive evaluations), they differ significantly in social cognitive abilities. This distinction highlights the need for caution when using acquired CSO as a model to explore the neurobiological basis of developmental CSO.

## Data Availability

The original contributions presented in the study are included in the article/**Supplementary Material**. Further inquiries can be directed to the corresponding author.
