# Supplementary material for: Cognitive profiles of paedophilic behaviour: a meta-analytic and systematic review of developmental vs acquired forms
Source: Front Psychiatry. 2025 Jun 9;16:1568244. doi: 10.3389/fpsyt.2025.1568244 (PMC12183301; doi:10.3389/fpsyt.2025.1568244)
Supplement: Supplementary file 1 [file SupplementaryFile1.docx]

**
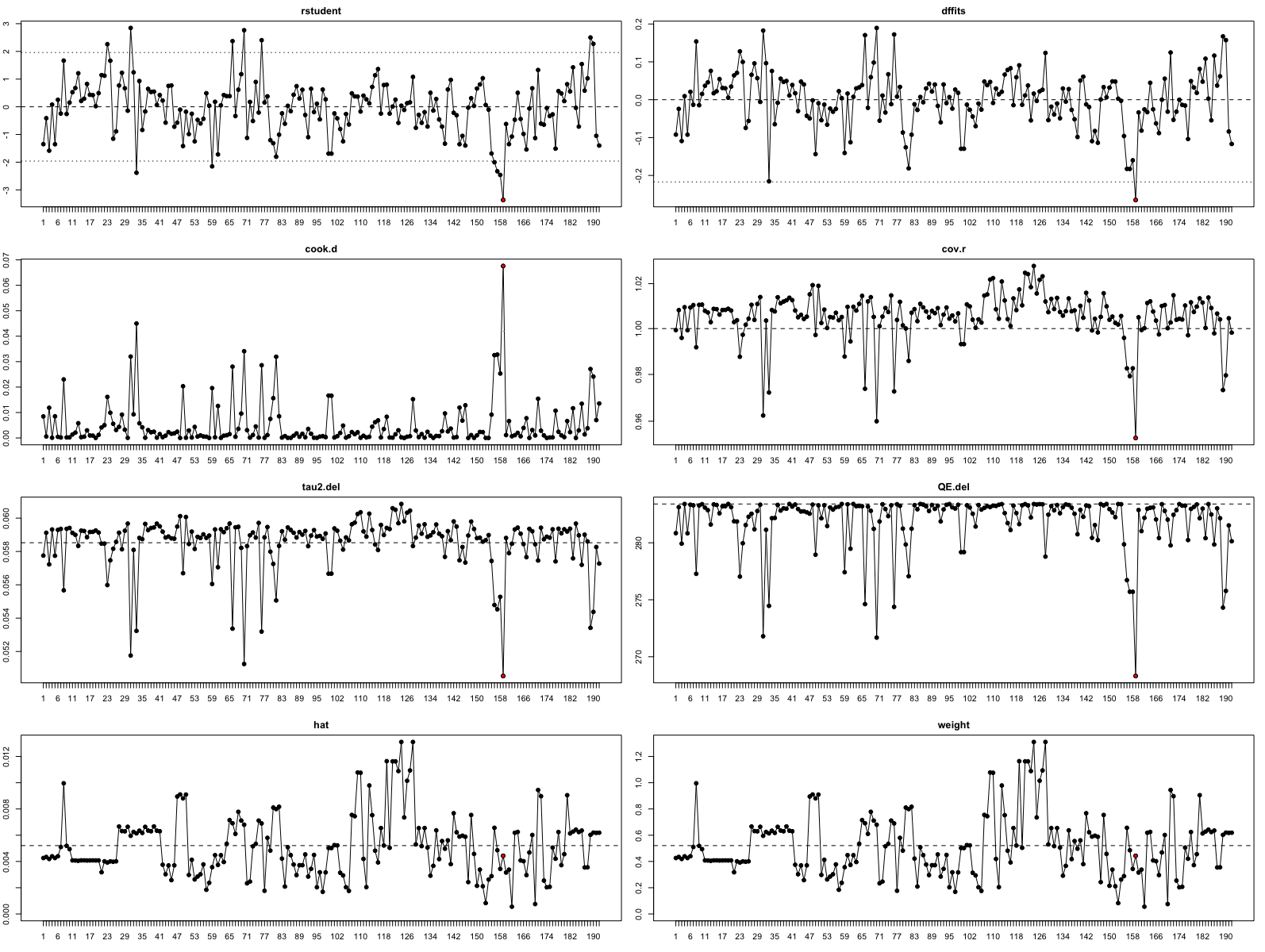
**

**Supplementary Figure 1.** Leave-one-out diagnostics by means of case-deletion diagnostics: rstudent: externally standardized residuals; DFFITS: difference in fitts; cook.d: Cook’s distances; cov.r: covariance ratios; tau2.del: estimates of Tau^2^ when each study is removed in turn; QE.del: test statistics for (residual) heterogeneity when each study is removed in turn; hat: diagonal elements of the hat matrix; weight: weights (in %) given to the observed outcomes during the model fitting.

**
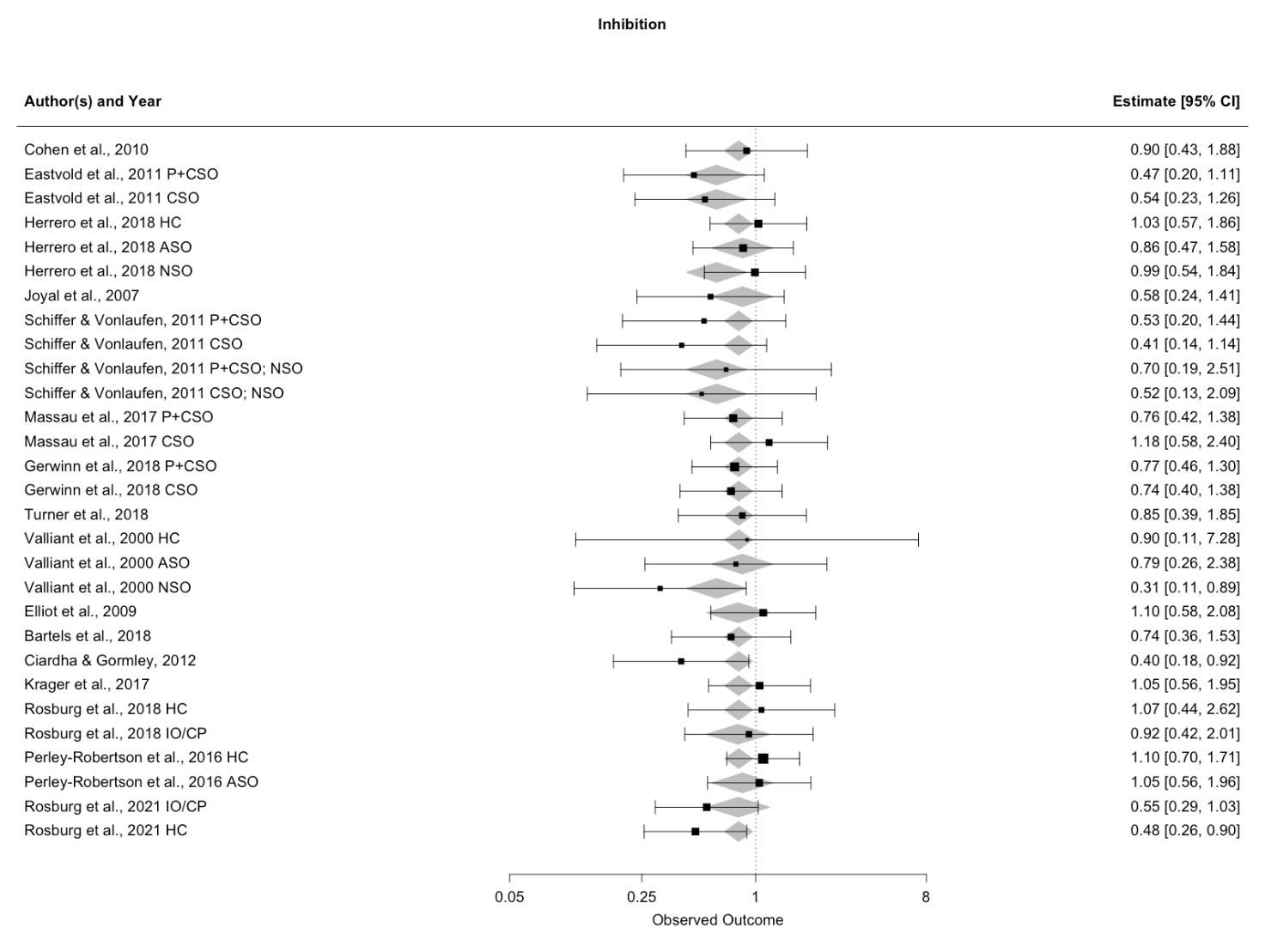
Supplementary Figure 2.** Forest plot with effect sizes of studies which investigated Inhibition abilities, included in the mixed-effects meta-regression model with Control group typologies as coefficients.


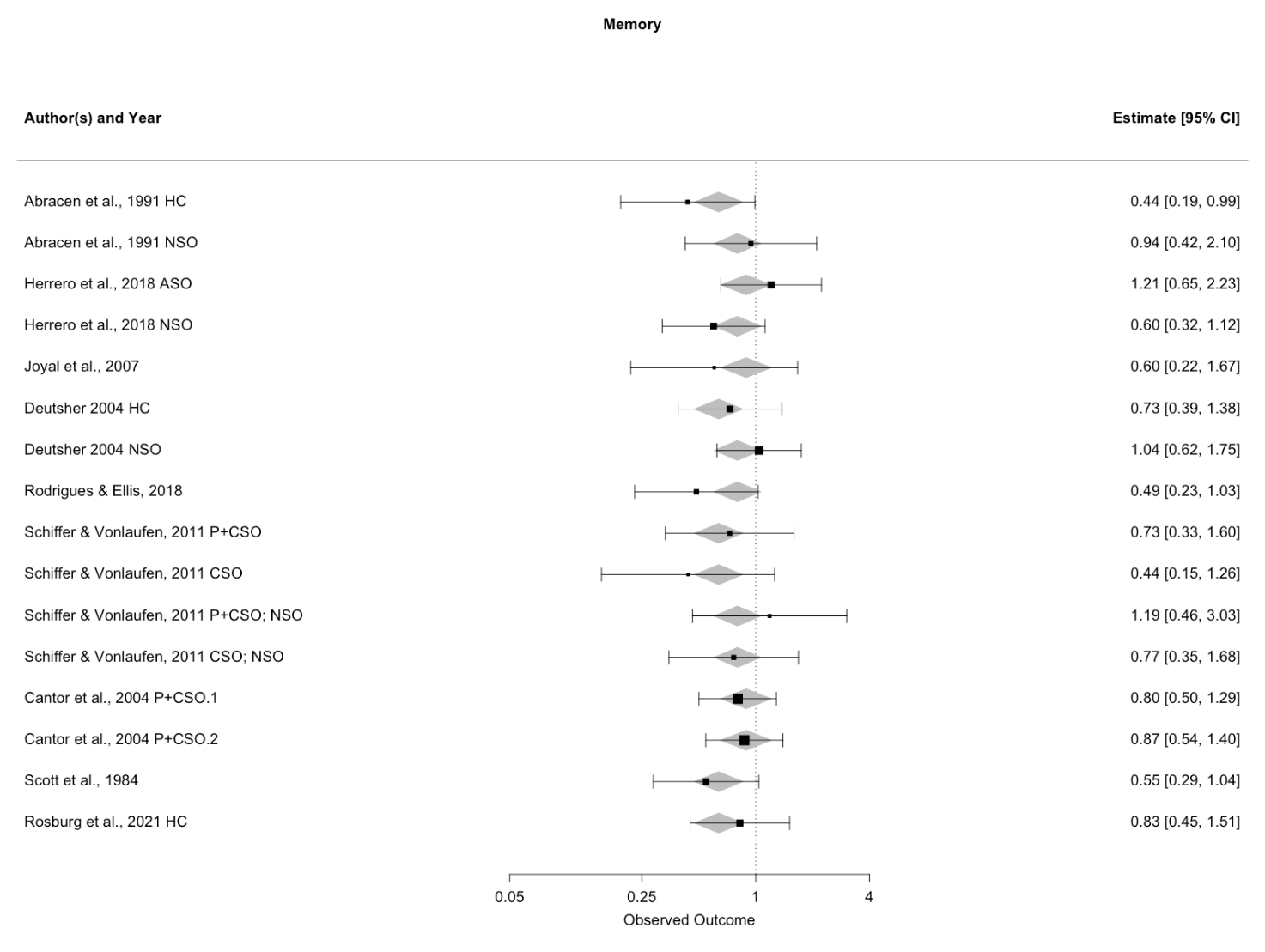


**Supplementary Figure 3.** Forest plot with effect sizes of studies which investigated Memory abilities, included in the mixed-effects meta-regression model with Control group typologies as coefficients.

**
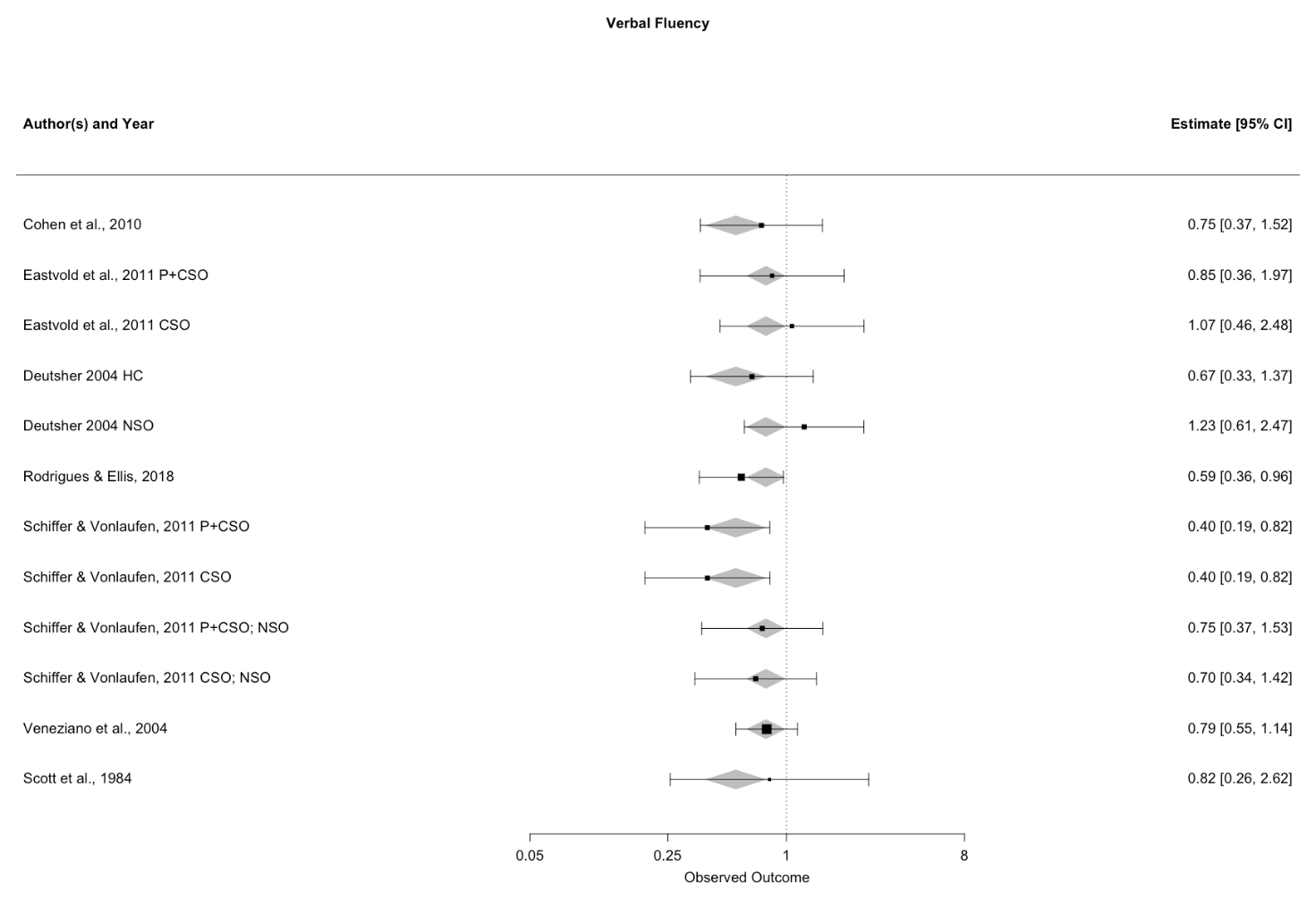
**

**Supplementary Figure 4.** Forest plot with effect sizes of studies which investigated Verbal Fluency abilities, included in the mixed-effects meta-regression model with Control group typologies as coefficients.
