# Supplementary material for: Cognitive profiles of paedophilic behaviour: a meta-analytic and systematic review of developmental vs acquired forms
Source: Front Psychiatry. 2025 Jun 9;16:1568244. doi: 10.3389/fpsyt.2025.1568244 (PMC12183301; doi:10.3389/fpsyt.2025.1568244)
Supplement: Supplementary file 2 [file Table1.docx]

| **Reference** | **Study group (n)** | **Controls (n)** | **Instrument** | **Cognitive domain** |
| --- | --- | --- | --- | --- |
| Abracen et al., 1991 | 12 CSO | 13 HC  12 NSO | TMT-B  CPM  WVLT | Set Switching  Planning  Memory |
| Azizian et al., 2016 | 114 P+CSO | 56 PNS | RBANS (Immediate memory; Delayed Memory)  RBANS (Visuospatial/Constructional)  RBANS (Attention)  RBANS (Language) | Memory  Planning  Attention  Verbal Fluency |
| Bartels et al., 2018 | 29 CSO | 25 HC | Fantasy-GNAT (Child block; Adult block)  Toy-GNAT (Child block; Adult block) | Inhibition/Impulsivity  Inhibition/Impulsivity |
| Becerra-Garcia & Egan, 2014 - Incest | 21 CSO | 28 HC | TMT-A  TMT-B  Digit Span (Forward; Backward) | Attention  Set Switching  Working Memory |
| Becerra-Garcia & Egan, 2014 – Non incest | 11 CSO | 28 HC | TMT-A  TMT-B  Digit Span (Forward; Backward) | Attention  Set Switching  Working Memory |
| Cantor et al., 2004 - Pedophilics | 47 P+CSO | 94 ASO | WAIS-R (Information; Similarities)  WAIS-R (Digit span; Arithmetic)  WAIS-R (Picture completion)  WAIS-R (Block design)  HVLT-R  BVMT-R | Verbal Semantic  Working Memory  Attention  Planning  Memory  Memory |
| Cantor et al., 2004 - Hebephilics | 161 P+CSO | 94 ASO | WAIS-R (Information; Similarities)  WAIS-R (Digit span; Arithmetic)  WAIS-R (Picture completion)  WAIS-R (Block design)  HVLT-R  BVMT-R | Verbal Semantic  Working Memory  Attention  Planning  Memory  Memory |
| Chaplin et al., 1995 | 15 CSO | 15 HC | HES  CSS | Social Cognition  Social Cognition |
| Ciardha & Gormley, 2012 | 23 CSO | 24 HC | Stroop Test (Congruous; Neutral; Incongruous) | Inhibition/Impulsivity |
| Cohen et al., 2010 | 50 P+CSO | 80 HC | COWA  MFFT  Porteus Mazes  Stroop Test (Colors; Words)  TMT-A  TMT-B  WCST | Verbal Fluency  Inhibition/Impulsivity  Inhibition/Impulsivity  Inhibition/Impulsivity  Attention  Set Switching  Set Switching |
| Deutsher 2004 | 25 CSO | 25 HC  25 NSO | WAIS-R (Arithmetic)  WAIS-R (Vocabulary)  WAIS-R (Block design)  WAIS-R (Picture completion)  RAVLT  RCF (Copy)  RCF (Organizational quality; Delay)  FAS  WCST | Working Memory  Verbal Semantic  Planning  Attention  Memory  Working Memory  Memory  Verbal Fluency  Set Switching |
| Eastvold et al., 2011 | 30 P+CSO  30 CSO | 29 NSO | DKEFS (TMT; Design Fluency Switch)  DKEFS (Color-Word interference)  DKEFS (Abstraction)  DKEFS (Verbal fluency; Design fluency)  DKEFS (Towers; Design move)  SILS (Abstraction)  SILS (Vocabulary)  WMS-III (Digit Span backward; Spatial Span backward)  WMS-III (Digit Span forward; Spatial Span forward)  WAIS-III (Information) | Set Switching  Inhibition/Impulsivity  Abstraction  Verbal Fluency  Planning  Abstraction  Verbal Semantic  Working Memory  Attention  Verbal Semantic |
| Elliot et al., 2009 | 526 CSO | 505 ICO | VEDS  CSCQ  IRI  BIS-11 | Social Cognition  Social Cognition  Social Cognition  Inhibition/Impulsivity |
| Franke et al., 2019 | 15 P+CSO | 15 UMC | TAP (Go/NoGo)  TAP (Allertness; Divided attention)  TAP (Tower of London) | Inhibition/Impulsivity  Attention  Planning |
| Gerwinn et al., 2018 | 83 P+CSO  30 CSO | 148 HC | WAIS-R (Vocabulary; Similarities)  WAIS-R (Block design; Matrix reasoning)  IRI  BIS-11 | Verbal Semantic  Planning  Social Cognition  Inhibition/Impulsivity |
| Gery et al., 2009 | 10 CSO | 10 NSO | EQS  IRI | Social Cognition  Social Cognition |
| Herrero et al., 2018 | 17 CSO | 32 HC  26 ASO  25 NSO | WAIS-R (Matrix reasoning)  WAIS-R (Similarities)  LMT  NLT  Simon Task | Planning  Verbal Semantic  Memory  Set Switching  Inhibition/Impulsivity |
| Joyal et al., 2007 | 12 CSO | 8 ASO | Stroop Test  Verbal Fluency  CVLT  WCST  TMT – B  ROCF (Copy)  ROCF (Delayed recall) | Inhibition/Impulsivity  Verbal Fluency  Memory  Set Switching  Set Switching  Working Memory  Memory |
| Joyal et al., 2020 | 39 CSO | 39 HC  41 NSO | WCST  IGT  Stop-It Test | Set Switching  Planning  Inhibition/Impulsivity |
| Krager et al., 2017 | 40 P+CSO | 40 HC | Go/NoGo | Inhibition/Impulsivity |
| Kruger & Schiffer, 2011 - Etero | 9 P+CSO | 14 HC | d2 ADT  WIP (General knowledge)  WIP (Commonality finding)  WIP (Completing images; Mosaic test)  CBTT  WCST | Attention  Verbal Semantic  Abstraction  Planning  Working Memory  Set Switching |
| Kruger & Schiffer, 2011 - Homo | 11 P+CSO | 14 HC | d2 ADT  WIP (General knowledge)  WIP (Commonality finding)  WIP (Completing images; Mosaic test)  CBTT  WCST | Attention  Verbal Semantic  Abstraction  Planning  Working Memory  Set Switching |
| Massau et al., 2017 | 45 P+CSO  19 CSO | 49 HC | SST  IST  IED  Stockings of Cambridge  Spatial Working Memory | Inhibition/Impulsivity  Inhibition/Impulsivity  Set Switching  Planning  Working Memory |
| Neutze et al., 2011 | 45 P+CSO | 42 CP | CIS | Social Cognition |
| Perley-Robertson et al., 2016 | 37 CSO | 74 HC  9 ASO | IS-7  BIS-11  GSCS  SSS-V  TSCS | Inhibition/Impulsivity  Inhibition/Impulsivity  Inhibition/Impulsivity  Inhibition/Impulsivity  Inhibition/Impulsivity |
| Rodrigues & Ellis, 2018 | 34 CSO | 32 NSO | Hayling Test  FAS  IGT  TMT-A  TMT-B  RCF (Copy)  RCF (Immediate recall)  RAVLT | Set Switching  Verbal Fluency  Planning  Attention  Set Switching  Working Memory  Memory  Memory |
| Rosburg et al., 2018 | 21 P+CSO | 21 HC  19 CP | Go/NoGo | Inhibition/Impulsivity |
| Rosburg et al., 2021 | 21 P+CSO | 21 HC  20 CP | ANT (Alerting)  ANT (Orienting)  CGT  Stroop  CVLT  Two-Back Task | Attention  Attention  Planning  Inhibition/Impulsivity  Memory  Working Memory |
| Schiffer & Vonlaufen, 2011 | 15 P+CSO  15 CSO | 17 HC  16 NSO | WCST  Go/NoGo  TMT-A  TMT-B  Regensburger Wortflüssigkeitstest  CBTT (Immediate recall; Delayed recall; Span recall)  LMT*  Tower of London | Set Switching  Inhibition/Impulsivity  Attention  Set Switching  Verbal Fluency  Memory  Memory  Planning |
| Schuler et al., 2019 | 85 P+CSO | 128 HC | IRI  MET | Social Cognition  Social Cognition |
| Scott et al., 1984 | 14 P+CSO | 31 HC | LNNB (Speech; Reading; Writing)  LNNB (Arithmetic)  LNNB (Memory)  LNNB (Intellectual) | Verbal Fluency  Working Memory  Memory  Planning |
| Suchy et al., 2009 | 20 P+CSO  20 CSO | 20 HC | WAIS-III (Information)  PIAT (Reading Comprehension)  Recognition Vocabulary | Verbal Semantic  Verbal Semantic  Verbal Semantic |
| Suchy et al., 2014 | 20 P+CSO  20 CSO | 20 HC | HRB (Finger tapping)  WAIS-III (Symbol search; Digit symbol coding) | Working Memory  Attention |
| Tierney & McCabe, 2001 | 36 CSO | 40 HC  36 ASO  36 NSO | MEES  Empat | Social Cognition  Social Cognition |
| Turner et al., 2018 | 58 CSO | 63 HC | Go/NoGo  IGT  DDT | Inhibition/Impulsivity  Planning  Inhibition/Impulsivity |
| Valliant et al., 2000 | 11 CSO | 20 HC  14 ASO  20 NSO | TNI-II  Porteus Mazes  SIV-PM  DIT | Planning  Inhibition/Impulsivity  Planning  Planning |
| Veneziano et al., 2004 | 60 CSO | 60 NSO | COWA  TMT-A  TMT-B  Tower of London  WCST | Verbal Fluency  Attention  Set Switching  Planning  Set Switching |

**Supplementary table 1. Summary and description of selected studies for the meta-analysis.** P+CSO: Individuals with a diagnosis of pedophilic disorder who committed sexual offenses against children; CSO: Individuals who committed sexual offenses against children; HC: Healthy controls; NSO: Individuals who committed nonsexual offenses; PNS: Individuals with paraphilia non otherwise specified; ASO: Individuals who committed sexual offenses against adults; ICO: Individuals who committed offenses against children through internet; UMC: Individuals diagnosed with an unspecified mental condition; CP: Individuals who made use of child pornography; TMT: Trail Making Test; CPM: Coloured Progressive Matrices; WVLT: Williams Verbal Learning Test; RBANS: Repeatable Battery for the Assessment of Neuropsychological Status; GNAT: Go/No-Go Association Task; WAIS-R: Wechsler Adult Intelligence Scale – Revised; HVLT-R: Hopkins Verbal Learning Test – Revised; BVMT-R: Brief Visuospatial Memory Test – Revised; HES: Hogan Empathy Scale; CSS: Childhood Sexuality Survey; COWA: Controlled Word Association Test; MFFT: Matching Familiar Figures Test; WCST: Wisconsin Card Sorting Test; RAVLT: Rey Auditory Verbal Learning Test; RFC: Rey Complex Figure; FAS: FAS Test; DKEFS: Delis Kaplan Executive Function Scale; SILS: Shipley Institute of Living Scales; WMS-III: Wechsler Memory Scale – III; WAIS-III: Wechsler Adult Intelligence Scale – III; VEDS: Victim Empathy Distortion Scale; CSCQ: Children and Sex Cognitions Questionnaire; IRI: Interpersonal Reactivity Index; BIS-11: Barratt Impulsivity Scale – 11; TAP: Test of Attentional Performance; EQS: Empathy Quotient Scale; LMT: Letter Memory Task; NLT: Number-Letter Task; CVLT: California Verbal Learning Test; ROCF: Rey-Osterrieth Complex Figure; IGT: Iowa Gambling Task; d2 ADT: d2 Attention Deficit Test; WIP: reduced version of the German Wechsler Adult Intelligence Scale; CBTT: Corsi Block-Tapping Test; SST: Stop Signal Task; IST: Information Sampling Task; IED: Intra/Extradimensional Set Shift Task; CIS: Child Identification Scale; IS-7: Impulsiveness Scale – 7; GSCS: Grasmick Self-Control Scale; SSS-V: Sensation Seeking Scale-V; TCSC: Tangney Self-Control Scale; ANT: Attention Network Task; CGT: Cambridge Gambling Task; LMT*: Logical Memory Task; MET: Multifaceted Empathy Test; LNNB: Luria-Nebraska Neuropsychological Battery; PIAT: Peabody Individual Achievement Test; HRB: Halstead-Reitan Battery; MEES: Mehrabian and Epstein Empathy Scale; DDT: Dame Dice Task; TNI-II: Test of Nonverbal Intelligence – II; SIV-PM: Survey of Interpersonal Values - Practical Mindedness; DIT: Defining Issues Test.

**References**

Abracen, J., O’carroll, R., & Ladha, N. (1991). Neuropsychological dysfunction in sex offenders? The Journal of Forensic Psychiatry, 2(2), 167–177.

Azizian, A., Hutton, S., Hughes, D., & Sreenivasan, S. (2016). Cognitional Impairment: Is There a Role for Cognitive Assessment in the Treatment of Individuals Civilly Committed Pursuant to the Sexually Violent Predator Act? Sexual Abuse, 28(8), 755–769.

Bartels, R. M., Beech, A. R., Harkins, L., & Thornton, D. (2017). Assessing Sexual Interest in Children Using the Go/No-Go Association Test. Sexual Abuse: A Journal of Research and Treatment, 107906321668611.

Becerra-García, J. A., & Egan, V. (2014). Neurocognitive Functioning and Subtypes of Child Molesters: Poorer Working Memory Differentiates Incestuous from Non-Incestuous Offenders. Psychiatry, Psychology and Law, 21(4), 585–590.

Cantor, J. M., Blanchard, R., Christensen, B. K., Dickey, R., Klassen, P. E., Beckstead, A. L., Blak, T., & Kuban, M. E. (2004). Intelligence, Memory, and Handedness in Pedophilia. Neuropsychology, 18(1), 3–14.

Chaplin, T. C., Rice, M. E., & Harris, G. T. (1995). Salient victim suffering and the sexual responses of child molesters. Journal of Consulting and Clinical Psychology, 63(2), 249–255.

Ciardha, C. Ó., & Gormley, M. (2012). Using a Pictorial-Modified Stroop Task to Explore the Sexual Interests of Sexual Offenders Against Children. Sexual Abuse, 24(2), 175–197.

Cohen, L. J., Nesci, C., Steinfeld, M., Haeri, S., & Galynker, I. (2010). Investigating the Relationship between Sexual and Chemical Addictions by Comparing Executive Function in Subjects With Pedophilia or Opiate Addiction and Healthy Controls. Journal of Psychiatric Practice, 16(6), 405–412.

Deutsher, M. (n.d.). A Neuropsychological Assessment of Adult Sex Offenders. 166.

Eastvold, A., Suchy, Y., & Strassberg, D. (2011). Executive Function Profiles of Pedophilic and Nonpedophilic Child Molesters. Journal of the International Neuropsychological Society, 17(2), 295–307.

Elliott, I. A., Beech, A. R., Mandeville-Norden, R., & Hayes, E. (2009). Psychological Profiles of Internet Sexual Offenders: Comparisons With Contact Sexual Offenders. Sexual Abuse, 21(1), 76–92.

Franke, I., Seipel, S., Vasic, N., Streb, J., Nigel, S., Otte, S., & Dudeck, M. (2019). Neuropsychological profile of pedophilic child sexual offenders compared with an IQ-matched non-offender sample – Results of a pilot study. International Journal of Law and Psychiatry, 64, 137–141.

Gerwinn, H., Weiß, S., Tenbergen, G., Amelung, T., Födisch, C., Pohl, A., Massau, C., Kneer, J., Mohnke, S., Kärgel, C., Wittfoth, M., Jung, S., Drumkova, K., Schiltz, K., Walter, M., Beier, K. M., Walter, H., Ponseti, J., Schiffer, B., & Kruger, T. H. C. (2018). Clinical characteristics associated with paedophilia and child sex offending – Differentiating sexual preference from offence status. European Psychiatry, 51, 74–85.

Gery, I., Miljkovitch, R., Berthoz, S., & Soussignan, R. (2009). Empathy and recognition of facial expressions of emotion in sex offenders, non-sex offenders and normal controls. Psychiatry Research, 165(3), 252–262.

Herrero, Ó., Escorial, S., & Colom, R. (2018). Rapists and Child Abusers Share Low Levels in Executive Updating, but Do not in Fluid Reasoning. The European Journal of Psychology Applied to Legal Context, 11(1), 1–7.

Joyal, C. C., Black, D. N., & Dassylva, B. (2007). The Neuropsychology and Neurology of Sexual Deviance: A Review and Pilot Study. Sexual Abuse, 19(2), 155–173.

Joyal, C. C., Tardif, M., & Spearson-Goulet, J.-A. (2020). Executive Functions and Social Cognition in Juveniles Who Have Sexually Offended. Sexual Abuse, 32(2), 179–202.

Kärgel, C., Massau, C., Weiß, S., Walter, M., Borchardt, V., Krueger, T. H. C., Tenbergen, G., Kneer, J., Wittfoth, M., Pohl, A., Gerwinn, H., Ponseti, J., Amelung, T., Beier, K. M., Mohnke, S., Walter, H., & Schiffer, B. (2017). Evidence for superior neurobiological and behavioral inhibitory control abilities in non-offending as compared to offending pedophiles: Response Inhibition in Pedophilia. Human Brain Mapping, 38(2), 1092–1104.

Kruger, T. H. C., & Schiffer, B. (2011). Neurocognitive and Personality Factors in Homo- and Heterosexual Pedophiles and Controls. The Journal of Sexual Medicine, 8(6), 1650–1659.

Massau, C., Tenbergen, G., Kärgel, C., Weiß, S., Gerwinn, H., Pohl, A., Amelung, T., Mohnke, S., Kneer, J., Wittfoth, M., Ristow, I., Schiltz, K., Beier, K. M., Ponseti, J., Walter, M., Kruger, T. H. C., Walter, H., & Schiffer, B. (2017). Executive Functioning in Pedophilia and Child Sexual Offending. Journal of the International Neuropsychological Society, 23(6), 460–470.

Neutze, J., Seto, M. C., Schaefer, G. A., Mundt, I. A., & Beier, K. M. (2011). Predictors of Child Pornography Offenses and Child Sexual Abuse in a Community Sample of Pedophiles and Hebephiles. Sexual Abuse, 23(2), 212–242.

Perley-Robertson, B., Helmus, L. M., Derkzen, D., & Serin, R. C. (n.d.). Do Sex Offenders Against Adults, Sex Offenders Against Children, and Non-sex Offenders Differ in Impulsivity? Sexual Offender Treatment, 21.

Rodriguez, M., & Ellis, A. (2018). The Neuropsychological Function of Older First-Time Child Exploitation Material Offenders: A Pilot Study. International Journal of Offender Therapy and Comparative Criminology, 62(8), 2357–2373.

Rosburg, T., Deuring, G., Boillat, C., Lemoine, P., Falkenstein, M., Graf, M., & Mager, R. (2018). Inhibition and attentional control in pedophilic child sexual offenders – An event-related potential study. Clinical Neurophysiology, 129(9), 1990–1998.

Rosburg, T., Pflueger, M. O., Mokros, A., Boillat, C., Deuring, G., Spielmann, T., & Graf, M. (2021). Indirect and neuropsychological indicators of pedophilia. *Sexual Abuse*, *33*(5), 579-605.

Schiffer, B., & Vonlaufen, C. (2011). Executive Dysfunctions in Pedophilic and Nonpedophilic Child Molesters. The Journal of Sexual Medicine, 8(7), 1975–1984.

Schuler, M., Mohnke, S., Amelung, T., Dziobek, I., Lemme, B., Borchardt, V., Gerwinn, H., Kärgel, C., Kneer, J., Massau, C., Pohl, A., Tenbergen, G., Weiß, S., Wittfoth, M., Waller, L., Beier, K. M., Walter, M., Ponseti, J., Schiffer, B., … Walter, H. (2019). Empathy in pedophilia and sexual offending against children: A multifaceted approach. Journal of Abnormal Psychology, 128(5), 453–464.

Scott, M. L., Cole, J. K., McKay, S. E., Golden, C. J., & Liggett, K. R. (1984). Neuropsychological Performance of Sexual Assaulters and Pedophiles. Journal of Forensic Sciences, 29(4), 11778J.

Suchy, Y., Eastvold, A. D., Strassberg, D. S., & Franchow, E. I. (2014). Understanding processing speed weaknesses among pedophilic child molesters: Response style vs. Neuropathology. Journal of Abnormal Psychology, 123(1), 273–285.

Suchy, Y., Whittaker, J. W., Strassberg, D. S., & Eastvold, A. (2009). Neurocognitive differences between pedophilic and nonpedophilic child molesters. Journal of the International Neuropsychological Society, 15(2), 248–257.

Tierney, D. W., & McCabe, M. P. (2001). An Evaluation of Self-Report Measures of Cognitive Distortions and Empathy among Australian Sex Offenders. Archives of Sexual Behavior, 25.

Turner, D., Laier, C., Brand, M., Bockshammer, T., Welsch, R., & Rettenberger, M. (2018). Response inhibition and impulsive decision-making in sexual offenders against children. Journal of Abnormal Psychology, 127(5), 471–481.

Valliant, P. M., Pottier, D., Gauthier, T., & Kosmyna, R. (n.d.). Moral Reasoning, Interpersonal Skills, and Cognition of Rapists, Child Molesters, and Incest Offenders. 9.

Veneziano, C., Veneziano, L., LeGrand, S., & Richards, L. (2004). Neuropsychological Executive Functions of Adolescent Sex Offenders and Nonsex Offenders. Perceptual and Motor Skills, 98(2), 661–674.
