# Supplementary material for: Cognitive profiles of paedophilic behaviour: a meta-analytic and systematic review of developmental vs acquired forms
Source: Front Psychiatry. 2025 Jun 9;16:1568244. doi: 10.3389/fpsyt.2025.1568244 (PMC12183301; doi:10.3389/fpsyt.2025.1568244)
Supplement: Supplementary file 3 [file Table2.docx]

| **Reference** | **Brain pathology (Anatomical localization)** | **Neurological symptoms** | **Social Cognition** (Ability to understand social and moral disvalue) | | | | **Inhibition abilities** | | | |
| --- | --- | --- | --- | --- | --- | --- | --- | --- | --- | --- |
|  |  |  | **Formal NPS Battery** | **Daily life** | ***Modus Operandi*** | **Interpretation** | **Formal NPS Battery** | **Daily life** | ***Modus Operandi*** | **Interpretation** |
| Lesniak, Szymusik, & Chrzanowski, 1972 | Glioma or meningioma  (Right frontal lobe) | Yes  (Small impairment, pathological reflexes of the legs) | He retained the ability to recognize the significance of his acts | n/a | Threatened to kill the victim if she revealed the fact | **Spared** | n/a | Hypersexuality (he was seen bathing nude with little children) | Enacted sexual inappropriate behavior in front of witnesses | **Impaired** |
| Regestein & Reich, 1978, patient 1 | Meningioma  (Right frontal craniotomy) | Yes  (Decreasing vision in left eye, postoperative neurological signs) | He showed no insight about his transgressions. He seems to be more worried by the momentary embarrassment they caused than their serious implications | Personality changes reported by the wife: indifference toward her | When caught by the wife, he denied any wrongdoing, but he was not able to explain his behavior | **Impaired** |  | Hypersexuality | Was found to act offensive behaviors with several children in his living room | **Impaired** |
| Miller, Cummings, McIntyre, Ebers, & Grode, 1986 | Astrocytoma  (Left brainstem from the pons to the midbrain, thalamus, hypothalamus) | Yes  (Urinary incontinence, Weber’s syndrome, Benedikt’s syndrome) | n/a | He frequently embarrassed his wife by showing pornographic pictures to visitors at their home | n/a | **Impaired** | n/a | n/a | He proposed to children in his neighbourhood | **Impaired** |
| Ortego, Miller, Itabashi, & Cummings, 1993 | MS  (Frontal lobes, basal ganglia, perithalamic septal region, hypothalamus) | Yes  (Symptoms of MS, left arm and leg weakness, paraesthesia, blurring of vision) | Not completed due to limited cooperation of the patient | Described as “surprisingly unconcerned” by her predicament. She never manifested concern for the legal consequences of her actions | The modus operandi suggests an impairment of judgement, as she asked an adolescent male to touch her breasts in front of children | **Impaired** | Not completed due to limited cooperation of the patient. The psychologist that observed the patient indicated that he had an altered impulse control | n/a | Some of her illegal sexual activities were performed in front of witnesses | **Impaired** |
| Dimitrov, Phipps, Zahn, & Grafman, 1999 | Firearm wound  (Right frontal ventromedial, anterior cingulate) | No | Major deficits in emotions and social behavior: socially inappropriate comments and inability to realise their inappropriateness. Impaired social adaptability: impaired ability to make social judgements; impaired social decision making. Low performance at the EETC | Post injury changes in personality and social behavior. Profound deficiency in how the patient related to others, no emotional reactions to events. He appeared unable to have a normal relationship and follow both ethical and legal social norms | n/a | **Impaired** | Severely impaired in BADS. Dis-inhibited in his social conversations, he did not stop talking until directed to perform. | Married three times (first time with an 18years old individual with drug addiction, which lasted 54 days; third wedding while he was still married to the second wife).  Gave unknown people large amounts of money on request | n/a | **Impaired** |
| Frohman, Frohman, & Moreault, 2002 | MS  (Periventricular, hypothalamic and brainstem lesions) | Yes  (Binocular diplopia, dysarthria, ataxia) | n/a | Poor judgement, which became even poorer  during the ten months following MS exacerbation | Prior to MS exacerbation he touched women, despite recognizing how inappropriate these actions were | **Spared before MS exacerbation Impaired after MS exacerbation (close to the time of the crime)** | Stroop test within the normal range before MS exacerbation | Impulsivity and hypersexuality (he masturbated more than 10 times per day) | He felt helpless to control his actions | **Impaired** |
| Burns & Swerdlow, 2003 | Hemangiopericytoma  (Anterior fossa skull base mass displacing the orbitofrontal lobe) | Yes  (Headache, balance problems, abnormal glabellar, snout and palmomental responses, urinary incontinence, ataxia) | n/a | n/a | He felt that his activities were unacceptable. He concealed the offenses because he was ashamed | **Spared** | Normal at Go/NoGo test | Despite his strong desire to avoid prison, he could not retrain himself from soliciting sexual favour from staff and other members of rehabilitative program | He reported that the “pleasure principle overrode his urge restraint” | **Impaired** |
| Solla, Floris, Tacconi, & Cannas, 2006 | Parkinson’s disease,  Hedonistic homeostatic dysregulation | Yes  (Drug induced dyskinesias) | n/a | n/a | He understood the moral disvalue of his behavior | **Spared** | n/a | n/a | He described his impulses as irrepressible | **Impaired** |
| Devinsky, Sacks, & Devinsky, 2010 | Ganglioglioma  (Right mesial temporal lobe) | Yes  (Tonic clonic seizures) | n/a | n/a | He felt ashamed and secretive about these activities | **Spared** | n/a | Hyperphagia and hypersexuality (including coprophilia); compulsion for pornography | n/a | **Impaired** |
| Mendez & Shapira, 2011, case 2 | bvFTD  (Atrophy of the frontal lobe, more extensive on the right) | Yes  (Saccadic pursuit of extraocular movements, brisk reflexes) | n/a | n/a | He displayed little concern or understanding of his behavior | **Impaired** | n/a | Dis-inhibited behavior, as producing ethnic slurs, compulsive acts, hyperorality | n/a | **Impaired** |
| Mendez & Shapira, 2011, case 3 | Alzheimer’s disease  (Bilateral temporal lobe atrophy, relatively intact frontal lobes) | No | Socially inappropriate behavior | n/a | n/a | **Not enough information available** | n/a | Excessively friendly with strangers. Socially inappropriate behavior (he pulled out his shirt to show a scar to strangers) | n/a | **Impaired** |
| Mendez & Shapira, 2011, case 4 | Vascular dementia  (Right globus pallidus lacune) | Yes  (Reflexes were brisk and symmetrical with upgoing toes and a positive glabellar tap) | n/a | n/a | n/a | **Not enough information available** | Perseverative and dis-inhibited behavior, with stimulus-bound behavior and poor impulse control | Uncontrollable sexual urges; marked dis-inhibition; more talkative, child-like, and prone to profane language. Often walked around naked | n/a | **Impaired** |
| Mendez & Shapira, 2011, case 5 | Parkinson’s disease, addiction to pramipexole (dopamine agonist) | Yes  (Cranial nerve evaluation: hypomimia and hypophonia; slow gait) | n/a | n/a | Fully aware of his behaviour and felt embarrassed and ashamed | **Spared** | n/a | Hypersexuality and marked impulsivity | n/a | **Impaired** |
| Mendez & Shapira, 2011, case 6 | Huntington’s disease  (Atrophy of putamen, caudate nuclei, hypometabolism of striatum) | Yes  (Dysarthria, uncontrollable movements in fingers, gait difficulties) | Constricted affect | n/a | Little insight into his behavior | **Impaired** | Dis-inhibition upon examination | Often behaved impulsively | Touched a child in front of her mother | **Impaired** |
| Mendez & Shapira, 2011, case 7 | Parkinson’s disease  (Right pallidotomy) | Yes  (Left sided rigidity, bradykinesia) | n/a | Complained intrusive sexual thought | He was ashamed of his behavior | **Spared** | n/a | Markedly hypersexual; complained urges that overwhelmed him | n/a | **Impaired** |
| Rainero et al., 2011 | bvFTD  (Asymmetric frontal atrophy) | n/a | Not aware of his sexual behavior | Social detachment, reduced insight, and awareness | n/a | **Impaired** | Verbal aggressivity | Hypersexuality and no other dis-inhibited behavior | n/a | **Not enough information available** |
| Fumagalli, Pravettoni, & Priori, 2015 | TBI  (Ventromedial right frontal lobe, left fronto temporal area) | Yes (Pathological Mingazzini test, left dysmetria) | Pathological social dilemma, excessive need to obtain social desirability | n/a | n/a | **Not enough information available** | Dysexecutive syndrome with disinhibition (BIS-11) | Irritable with uncontrollable emotional reactions; disinhibition | n/a | **Impaired** |
| Gilbert & Vranic, 2015; Gilbert, Vranic, & Viana, 2016 | Glioblastoma multiforme  (Left frontal lobe) | Yes  (Epileptic symptoms) | n/a | n/a | n/a | **Not enough information available** | n/a | n/a | n/a | **Not enough information available** |
| Sartori, Scarpazza, Codognotto, & Pietrini, 2016; Scarpazza, Pellegrini, Pietrini, & Sartori, 2018 | Clivus Chordoma  (Hypothalamus, orbitofrontal cortex) | Yes  (Tunnel vision, diplopia, asymmetrical brisk reflexes) | Impaired identification of behavioral violation in social situations test | Upon arrest, he asked his receptionist to cancel the patients’ appointments for that afternoon but to keep the ones for the day after; he started to watch pornography worriless of being seen by his wife | Absence of masking (left the door open) | **Impaired** | Defective performance at Hayling test | Stole from souvenir shops; easily frustrated | Absence of masking (left the door open) | **Impaired** |
| Scarpazza, Pennati, & Sartori, 2018, case 1 | bvFTD  (Bilateral atrophy of the frontal lobes) | Yes (Dysexecutive syndrome) | Deficit in counterfactual thinking | Upon arrest, he was unaware of the severity of his behavior and moral disvalue; affective indifference; scarce empathy | Enacting offensive behaviors in public places (i.e., cinema). | **Impaired** | Dis-inhibition at formal NPS assessment | He manifested irrational and impulsive behavior (e.g., bought expired food); verbally aggressive; hyperphagia and hypersexuality; spent all his savings; kleptomania | Enacting offensive behaviors in public places (i.e., cinema) | **Impaired** |
| Scarpazza, Pennati, et al., 2018, case 2 | Meningothelial meningioma  (Frontal and parietal right lobes) | Yes (Spatiotemporal disorientation) | Not tested | Not investigated | Unclear | **Not enough information available** | Clear deficit in inhibition of automatic answers and behaviors | n/a | Offenses enacted in front of a primary school garden | **Impaired** |

**Supplementary Table 2. Social Cognition and Impulse Control analysis in cases of acquired pedophilia**. BADS: Behavioral assessment of dysexecutive syndrome; BIS-11: Barratt Impulsiveness Scale-11; bvFTD: Behavioral Variant of Frontotemporal dementia; EECT: Emotional Empathic Tendency Scale; MS: Multiple Sclerosis; NPS: Neuropsychological; TBI: Traumatic Brain Injury; n/a: the information is not available/present within the original paper.

**References (already present in the main manuscript)**

Burns, J. M., & Swerdlow, R. H. (2003). Right orbitofrontal tumor with pedophilia symptom and constructional apraxia sign. *Arch Neurol, 60*(3), 437-440. doi:10.1001/archneur.60.3.437

Devinsky, J., Sacks, O., & Devinsky, O. (2010). Kluver-Bucy syndrome, hypersexuality, and the law. *Neurocase, 16*(2), 140-145. doi:10.1080/13554790903329182

Dimitrov, M., Phipps, M., Zahn, T. P., & Grafman, J. (1999). A thoroughly modern Gage. *Neurocase, 5*, 345-354.

Frohman, E. M., Frohman, T. C., & Moreault, A. M. (2002). Acquired sexual paraphilia in patients with multiple sclerosis. *Arch Neurol, 59*(6), 1006-1010. doi:10.1001/archneur.59.6.1006

Fumagalli, M., Pravettoni, G., & Priori, A. (2015). Pedophilia 30 years after a traumatic brain injury. *Neurol Sci, 36*(3), 481-482. doi:10.1007/s10072-014-1915-1

Gilbert, F., & Vranic, A. (2015). Paedophilia, Invasive Brain Surgery, and Punishment. *J Bioeth Inq, 12*(3), 521-526. doi:10.1007/s11673-015-9647-3

Gilbert, F., Vranic, A., & Viana, J. N. M. (2016). Acquired Pedophilia and Moral Responsibility. *ajob Neuroscience, 7*(4), 209-2011.

Lesniak, R., Szymusik, A., & Chrzanowski, R. (1972). Case report: Multidirectional disorders of sexual drive in a case of brain tumour. *Forensic Sci, 1*(3), 333-338. doi:10.1016/0300-9432(72)90031-3

Mendez, M., & Shapira, J. S. (2011). Pedophilic behavior from brain disease. *J Sex Med, 8*(4), 1092-1100. doi:10.1111/j.1743-6109.2010.02172.x

Miller, B. L., Cummings, J. L., McIntyre, H., Ebers, G., & Grode, M. (1986). Hypersexuality or altered sexual preference following brain injury. *J Neurol Neurosurg Psychiatry, 49*(8), 867-873. doi:10.1136/jnnp.49.8.867

Ortego, N., Miller, B. L., Itabashi, H., & Cummings, J. L. (1993). Altered sexual behavior with multiple sclerosis: a case report. *Neuropsychiatry: Neuropsychology and Behavioral Neurology, 6*(4), 260-264.

Rainero, I., Rubino, E., Negro, E., Gallone, S., Galimberti, D., Gentile, S., . . . Pinessi, L. (2011). Heterosexual pedophilia in a frontotemporal dementia patient with a mutation in the progranulin gene. *Biol Psychiatry, 70*(9), e43-44. doi:10.1016/j.biopsych.2011.06.015

Regestein, Q. R., & Reich, P. (1978). Pedophilia occurring after onset of cognitive impairment. *J Nerv Ment Dis, 166*(11), 794-798. doi:10.1097/00005053-197811000-00007

Sartori, G., Scarpazza, C., Codognotto, S., & Pietrini, P. (2016). An unusual case of acquired pedophilic behavior following compression of orbitofrontal cortex and hypothalamus by a Clivus Chordoma. *J Neurol, 263*(7), 1454-1455. doi:10.1007/s00415-016-8143-y

Scarpazza, C., Pellegrini, S., Pietrini, P., & Sartori, G. (2018). The role of neuroscience in the evaluation of mental insanity: On the controversies in Italy: Comment on “On the stand. Another episode of neuroscience and law discussion from Italy”. *Neuroethics, 11*(1), 83-95.

Scarpazza, C., Pennati, A., & Sartori, G. (2018). Mental Insanity Assessment of Pedophilia: The Importance of the Trans-Disciplinary Approach. Reflections on Two Cases. *Front Neurosci, 12*, 335. doi:10.3389/fnins.2018.00335

Solla, P., Floris, G., Tacconi, P., & Cannas, A. (2006). Paraphilic behaviours in a parkinsonian patient with hedonistic homeostatic dysregulation. *Int J Neuropsychopharmacol, 9*(6), 767-768. doi:10.1017/S1461145705006437
